# Comparative Genomics of *Lentilactobacillus parabuchneri* isolated from dairy, KEM complex, Makgeolli, and Saliva Microbiomes

**DOI:** 10.1186/s12864-022-09053-y

**Published:** 2022-12-05

**Authors:** Ismail Gumustop, Fatih Ortakci

**Affiliations:** BioEngineering Department, Faculty of Life and Natural Sciences, Abdullah Gul University, Kayseri, Turkey

**Keywords:** *Lentilactobacillus parabuchneri*, Comparative genomics, CRISPR-Cas, Prophage, Food spoilage, Histamine

## Abstract

**Background:**

*Lentilactobacillus parabuchneri* is of particular concern in fermented food bioprocessing due to causing unwanted gas formation, cracks, and off-flavor in fermented dairy foods. This species is also a known culprit of histamine poisonings because of decarboxylating histidine to histamine in ripening cheese. Twenty-eight genomes in NCBI GenBank were evaluated via comparative analysis to determine genomic diversity within this species and identify potential avenues for reducing health associated risks and economic losses in the food industry caused by these organisms.

**Result:**

Core genome-based phylogenetic analysis revealed four distinct major clades. Eight dairy isolates, two strains from an unknown source, and a saliva isolate formed the first clade. Three out of five strains clustered on clade 2 belonged to dairy, and the remaining two strains were isolated from the makgeolli and Korean effective microorganisms (KEM) complex. The third and fourth clade members were isolated from Tete de Moine and dairy-associated niches, respectively. Whole genome analysis on twenty-eight genomes showed ~ 40% of all CDS were conserved across entire strains proposing a considerable diversity among *L. parabuchneri* strains analyzed. After assigning CDS to their corresponding function, ~ 79% of all strains were predicted to carry putative intact prophages, and ~ 43% of the strains harbored at least one plasmid; however, all the strains were predicted to encode genomic island, insertion sequence, and CRISPR-Cas system. A type I-E CRISPR-Cas subgroup was identified in all the strains, with the exception of DSM15352, which carried a type II-A CRISPR-Cas system. Twenty strains were predicted to encode histidine decarboxylase gene cluster that belongs to not only dairy but also saliva, KEM complex, and unknown source. No bacteriocin-encoding gene(s) or antibiotic resistome was found in any of the *L. parabuchneri* strains screened.

**Conclusion:**

The findings of the present work provide in-depth knowledge of the genomics of *L. parabuchneri* by comparing twenty-eight genomes available to date. For example, the hdc gene cluster was generally reported in cheese isolates; however, our findings in the current work indicated that it could also be encoded in those strains isolated from saliva, KEM complex, and unknown source. We think prophages are critical mobile elements of *L. parabuchneri* genomes that could pave the way for developing novel tools to reduce the occurrence of this unwanted species in the food industry.

**Supplementary Information:**

The online version contains supplementary material available at 10.1186/s12864-022-09053-y.

## Background

The *Lentilactobacillus* (*L*) species *parabuchneri* was described by [1] and associated with various ecological niches, for example, cheese, silage, human saliva, brewery yeasts, and ropy beer [2–5]. *L. parabuchneri* is Gram (+), non-motile, catalase and Voges-Proskauer negative, facultative anaerobe, rod-shaped (~ 0.9 × 3 μm), and appears as single, pairs or short rod chains under the microscope (Farrow et al. 1988). *L. parabuchneri* grows at 15 °C but not at 45 °C [6]; however, some strains can grow at 5 °C [1]. It produces CO_2_ from L-glucose, presumably, lactic acid is biosynthesized from L-arabinose, D-raffinose, ribose, sucrose, D-fructose, D-glucose, gluconate, galactose, melibiose, melezitose, and maltose. However, some *L. parabuchneri* strains can utilize lactose (the primary carbon source available in milk) and convert it into acid [1]. The five-carbon and six-carbon sugar fermentation in *L. parabuchneri* occurs through the pentose phosphate pathway (PPP) because of the organism’s obligatory heterofermentative lifestyle [7]. *L. parabuchneri* produces ornithine and 1,2-propanediol from L-arginine and lactic acid, respectively. *L. parabuchneri* is also known to decarboxylase histidine into histamine, a strain-dependent trait of this species [8]. Previous studies reported that *L. parabuchneri* can convert lactic acid to carbon dioxide, acetate, or 1,2-propanediol anaerobically, a valuable metabolic trait combating pH decrease in the cytoplasm due to large amounts of lactate accumulation [8, 9]. The ability of *L. parabuchneri* to degrade lactate to acetate makes this species instrumental in certain fermentation processes, particularly silage stabilization [10–12]. Even though the lactate conversion trait is helpful for certain bioprocesses, it can be harmful to food fermentations [10]. For example, lactate degradation causes a pH increase and favors the growth of acid-sensitive spoilage organisms. Moreover, the formation of carbon dioxide somewhat contributes to unwanted gas production in the ripening cheese [13]. The production of ornithine, ammonia, CO_2_, and ATP from L-arginine is another unique metabolic attribute of *L. parabuchneri*, protecting against acid stress conditions by maintaining a favorable pH in the cytoplasm. Again, the formation of ammonia and CO_2_ leads to pH elevation and undesirable gas occurrence in cheese resulting in unwanted metabolites that ultimately cause food spoilage [13]. Nevertheless, *L. parabuchneri* ferments C6 sugars through the pentose phosphate pathway, which releases a mole of CO_2_ upon utilizing one mole of hexose sugars available in ripening cheese that perhaps contributes to unwanted gassiness [14–16].

Lactic acid bacteria (LAB) are heavily accustomed to specific environmental microniches and carry smaller genomes as opposed to other bacteria as a result of genome reduction, which leads to the preservation of a small number of critical genes necessary for niche-specific survivability [10, 17]. Despite their smaller genomes, LAB should maintain the ability to rapidly and continuously adapt to its respective environment, presumably via transduction by bacteriophage infection and horizontal gene transfer (HGT) [18].

Bacteriophages replicate using the bacterial host’s cellular machinery. While certain bacteriophages are lytic (i.e., lyse their host upon replication), others could undergo a lysogenic life cycle. In lysogeny, the genome of the bacteriophage integrates into the host chromosome in the form of a prophage, which is later replicated as the host duplicates. The prophage can be induced in response to certain environmental conditions resulting in the initiation of a lytic replication [19]. Later, prophage DNA is excised from the microbial genome and transformed into complete and intact phage particles that facilitate HGT [20, 21]. CRISPR (Clustered Regularly Interspaced Short Palindromic Repeats) and CRISPR-associated genes help bacteria to defend themselves against bacteriophages by acquiring and integrating short and repetitive viral sequences into their genomes. These repeats could be located on both chromosomal and plasmid DNA and are separated via spacer elements [22].

Even though *L. parabuchneri* is diverse in isolation source and a problematic species causing food spoilage and histamine poisonings, there are few studies overall for this species. The shortage of studies on *L. parabuchneri* has resulted in rather limited knowledge regarding genomic diversity at the strain level. To fully leverage the genomic potential of *L. parabuchneri* and understand the framework of strategies to control this species unwanted growth in food systems, we first should evaluate genetic species diversity. Therefore, the present work aimed to fill this gap in the literature by comparing all the genomes available to date in NCBI GenBank and proposing genome-guided solutions for strains of interest.

## Results

### Genomic traits

Thirty *L. parabuchneri* genomes from NCBI GenBank (https://www.ncbi.nlm.nih.gov/genome/browse/#!/prokaryotes/41166/) were used in this study. Two strains (i.e., FAM23167 and VRA_07sq_f) showing incomplete assemblies, as revealed from BUSCO analysis (Fig. S1), were eliminated from further comparative genomic analysis. Table 1 shows twenty-eight *L. parabuchneri* strains isolated from various ecological niches, including cheese, milk, saliva, KEM complex, makgeolli, and unknown source, representing a broad ecological and genetic diversity within the species. Annotations of twenty-eight genome assemblies resulted in genome sizes between 2.53 and 2.80 Mbp (2.64 Mbp average) (Table 1). The GC content of each genome slightly varied and ranged from 43.20 to 43.60% (43.39% average), which is consistent with reference strain KEM (43.6%). The coding sequences in each genome ranged between 2325 and 2675, with a mean of ~ 2492. The mean protein-coding sequences encoding putative prophage and CRISPR loci were calculated at 1.5% and 1.9%, respectively.

**Table 1:** Genomic features of twenty-eight *L. parabuchneri* strains were analyzed in this study

Strain	Isolation Source	Accession Number	Sequencing Technology	Size (Mb)	GC (%)	CDS	tRNA	rRNA	Intact Prophages	Plasmid
DSM 15352	NA	GCA_001437335.1	Illumina MiSeq; Illumina HiSeq	2.6	43.5	2434	54	7	0	1
DSM 5707	saliva	GCA_001435315.1	Illumina MiSeq; Illumina HiSeq	2.57	43.4	2386	54	3	0	1
FAM21731	Emmental	GCA_001922025.1	PacBio	2.73	43.5	2566	62	15	4	2
FAM21809	Tete de Moine	GCA_002095795.1	Illumina HiSeq	2.57	43.5	2462	60	9	3	0
FAM21823	Mont Soleil	GCA_002095615.1	Illumina HiSeq	2.67	43.3	2539	60	7	1	1
FAM21829	Emmental	GCA_002095645.1	Illumina HiSeq	2.74	43.3	2616	60	7	2	1
FAM21834	Tilsit	GCA_002095655.1	Illumina HiSeq	2.76	43.3	2663	60	7	2	1
FAM21835	Tilsit	GCA_002095755.1	Illumina HiSeq	2.67	43.5	2527	63	12	4	0
FAM21838	Swiss Alpine cheese	GCA_002095635.1	Illumina HiSeq	2.54	43.5	2419	59	8	0	0
FAM23163	Tete de Moine	GCA_002095835.1	Illumina HiSeq	2.57	43.5	2386	60	7	1	0
FAM23164	Tete de Moine	GCA_002095845.1	Illumina HiSeq	2.7	43.3	2626	60	9	2	0
FAM23165	Tete de Moine	GCA_002095695.1	Illumina HiSeq	2.71	43.3	2618	60	9	3	0
FAM23166	Tete de Moine	GCA_002095725.1	Illumina HiSeq	2.71	43.3	2628	60	7	3	0
FAM23168	Tete de Moine	GCA_002095715.1	Illumina HiSeq	2.57	43.5	2385	60	7	1	0
FAM23169	Tete de Moine	GCA_002095825.1	Illumina HiSeq	2.8	43.2	2675	60	8	4	1
FAM23279	milk	GCA_002095895.1	Illumina HiSeq	2.61	43.3	2478	59	9	0	0
FAM23280	milk	GCA_002095905.1	Illumina HiSeq	2.56	43.4	2419	59	8	0	0
FAM23281	milk	GCA_002095915.1	Illumina HiSeq	2.62	43.3	2479	59	8	2	0
FAM23282	milk	GCA_002095765.1	Illumina HiSeq	2.61	43.3	2488	59	8	2	0
IPLA 11117	cheese	GCA_001687155.1	Illumina HiSeq	2.77	43.2	2624	58	7	1	0
IPLA 11122	cheese	GCA_001677035.1	Illumina HiSeq	2.56	43.5	2398	58	7	2	0
IPLA 11150	cheese	GCA_001687145.1	Illumina HiSeq	2.69	43.3	2544	58	7	1	1
IPLA11125	cheese	GCA_019266025.1	Illumina	2.58	43.5	2396	58	7	1	0
IPLA11129	cheese	GCA_019266005.1	Illumina	2.58	43.5	2396	60	7	1	1
IPLA11151	cheese	GCA_019265985.1	Illumina	2.66	43.4	2504	60	7	1	1
KEM	KEM complex	GCA_014879295.1	Illumina GAIIx	2.53	43.6	2325	60	15	1	0
NBRC 107865	NA	GCA_001591885.1	Illumina HiSeq 1000	2.55	43.4	2380	36	3	0	1
NSMJ16	Makgeolli (Korean traditional alcoholic beverage)	GCA_014905035.1	Illumina HiSeq; PacBio RSII	2.61	43.5	2413	61	15	0	3

### Comparative genomics

The twenty-eight *L. parabuchneri* genomes were put forth in a comparative genomic analysis. Twenty-eight strains, including the reference genome of KEM in the NCBI GenBank public database, were selected for phylogenetic analysis using nucleotide sequence alignment of the highly granulated phosphoglucomutase gene (Fig. S2). Four distinct clades were formed. The first clade consisted of cheese isolates of IPLA11151, IPLA11150, IPLA11125, IPLA11122, IPLA11117, and a single isolate from an unknown source (NBRC107865). Second clade members were isolated from cheese (FAM23168, FAM23163, and FAM21829), saliva (DSM5707), and an unknown source (DSM15352). Third clade members contained the reference strain KEM in addition to cheese (IPLA11129, FAM23169, FAM21834, FAM21823, and FAM21731) and makgeolli (NSMJ16). The remaining strains that formed the fourth clade were primarily isolated from dairy (Fig. S2).

**Fig. 1:**
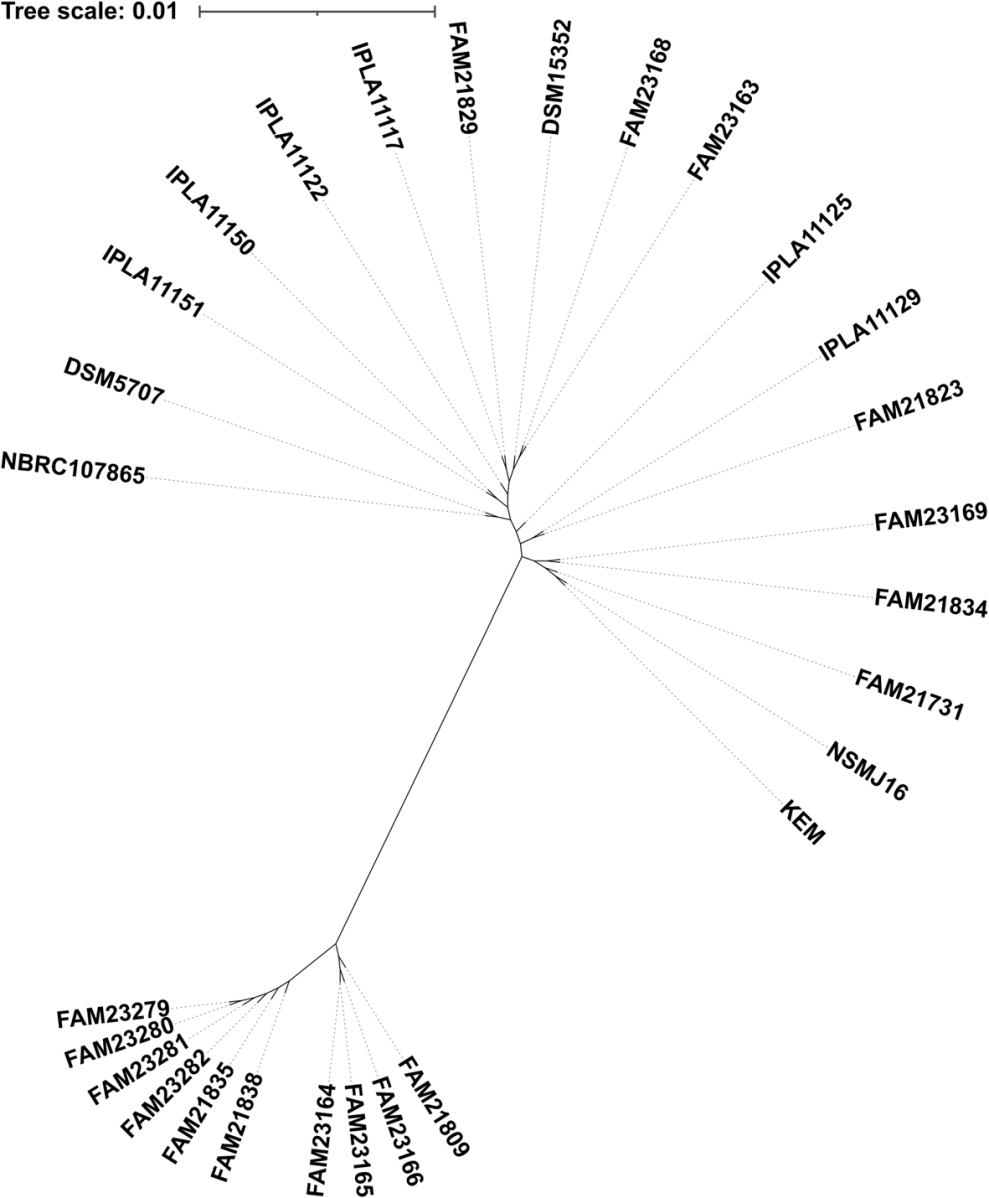
Neighbor-joining unrooted phylogenetic tree based on core genome alignment obtained using Roary [23] and FastTree [24]

Secondly, we constructed a core genome-based neighbor-joining unrooted phylogenetic tree using Roary [23] and FastTree [24] (Fig. 1). Four major clades were identified. The first clade consisted of isolates of dairy strains (IPLA11151, IPLA11150, IPLA11122, IPLA11117, FAM21829, FAM23168, FAM23163, IPLA11125, IPLA11129, and FAM21823) in addition to saliva isolate (DSM5707), and two strains from an unknown source (NBRC107865 and DSM15352). Although the second clade members mainly were isolated from dairy (FAM23169, FAM21834, and FAM21731), makgeolli isolate (NSMJ16) and the reference strain (KEM) lay on this clade. Members of the third clade, FAM21809, FAM23166, FAM23165, and FAM23164, were only isolated from Tete de Moine cheese. The remaining six strains forming the last clade were also dairy isolates. The closest neighbor to the reference strain was NSMJ16. Thirdly, we performed a whole genome-based phylogenetic tree using TYGS [25] to understand pan- vs. core genome-based differences in phylogenies. Three clades were identified (Fig. S3), with the first member clade containing FAM21809, FAM23164, FAM23165, and FAM23166 isolated from Tete de Moine cheese. Similarly, second-clade members (FAM23282, FAM21835, FAM21838, FAM23279, FAM23280, and FAM23281) were isolated from cheese or milk. The eighteen strains, including the reference genome, formed the third clade. The closest neighbor to KEM was found to be NSMJ16 (Fig. S3). When clustered by gene absence/presence matrix, three distinct groups emerged as well (Fig. S4). While group 1 and group 2 only comprised dairy-associated strains, group 3 contained eighteen strains isolated from dairy, saliva, KEM complex, makgeolli, and unknown source.

**Fig. 2:**
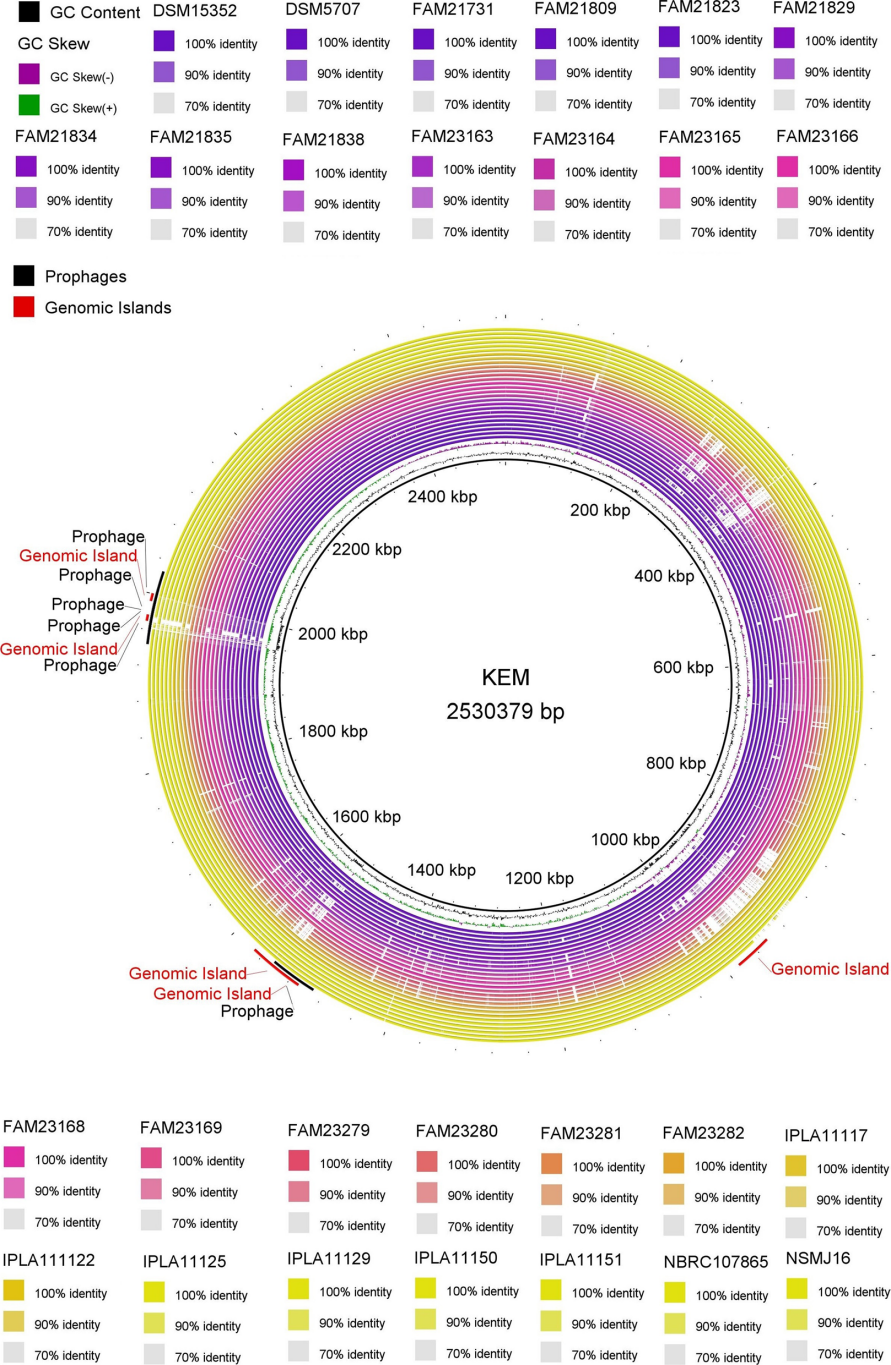
Whole-genome-based BLAST comparison of 27 *L. parabuchneri* strains against reference strain KEM

BRIG (BLAST Ring Image Generator) was utilized for performing a comparative whole genome analysis of twenty-seven *L. parabuchneri* strains against the reference genome KEM (Fig. 2). In general, when we compared the putative CDS of all strains against the reference genome KEM, high percentage identity was evident with 70 to 100% BLAST identity range as shown in Fig. 2. The decreasing GC percentage and lower BLAST identity identified three main gaps in coverage. Although the first gap in coverage at the five o’clock direction consisted of a single genomic island, the second gap in coverage at the seven o’clock direction contained two genomic islands and a single prophage. The last gap in coverage at nine-thirty o’clock is composed of a couple of genomic islands and five intact prophages (Fig. 2, Table S1-S2).

### Pan- and core genome analysis

**Fig. 3:**
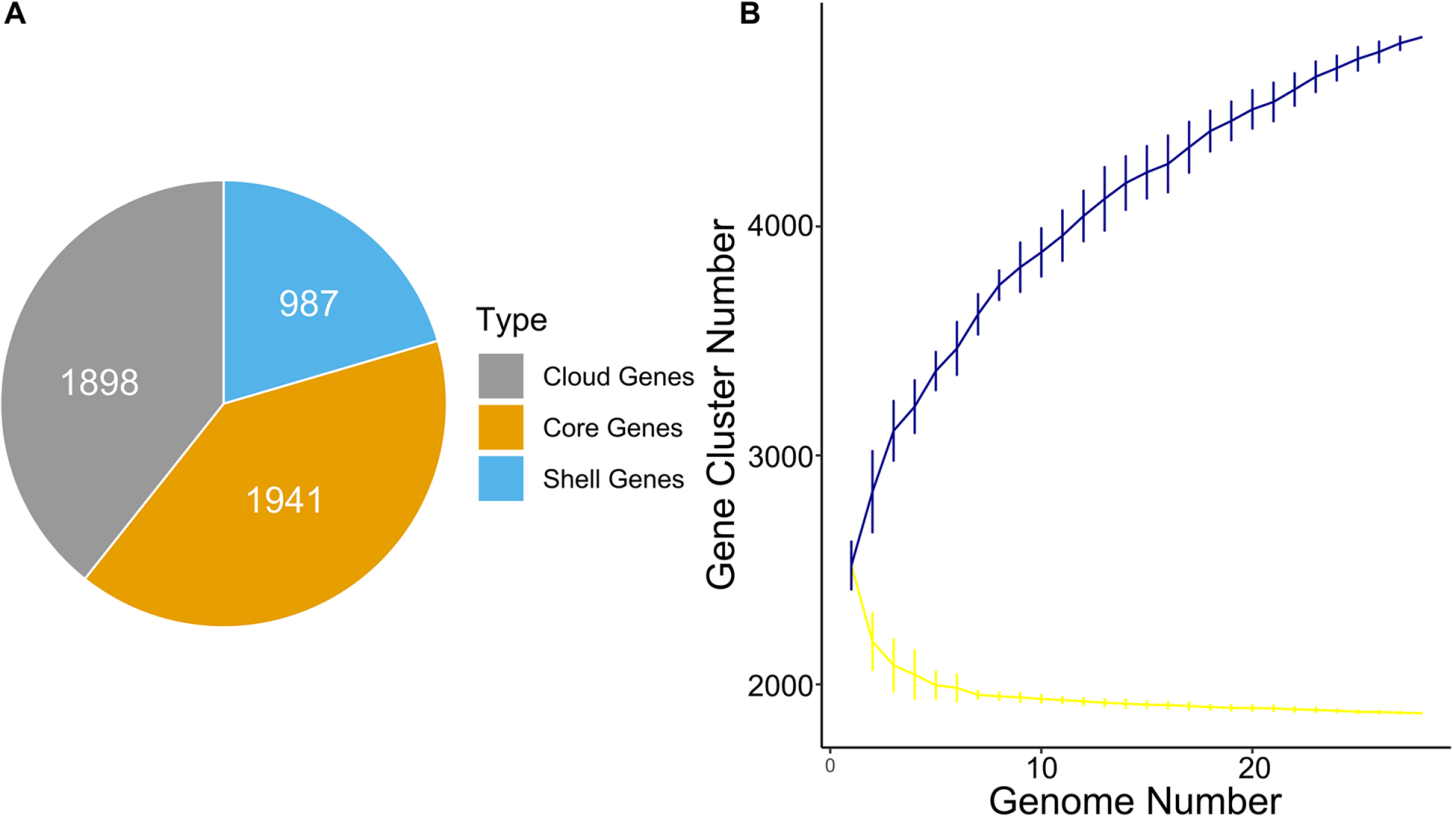
**A** Distributions of coding sequences found in twenty-eight *L. parabuchneri* pan-genomes: Core genes (orange), shell genes (blue), and cloud genes (grey) in the chromosome. **B** Estimation of the pan-genome (blue) and the core genome (yellow) of twenty-eight *L. parabuchneri* strains by including genomes one by one. R programming language [27] and ggplot2 [28] package were used to plot the graph

Genomic conservation analysis across twenty-eight isolates regarding pan- and core genomes revealed that 40.2% of the entire genes were conserved within 95% BLASTP identity (Fig. 3 A). Of the 4826 total coding sequences, 1941 genes were shared across 28 strains which correspond to the core genome. The shell genes contained 20.45% of the entire CDS, whereas cloud genes represented 39.3% of total coding sequences implying phenotypic differences among *L. parabuchneri* strains [26]. For further insights, we performed random subsampling for constructing trend lines of each strain’s pan- and core genome (Fig. 3B). The core genome size was near to flatline at the twenty-eighth strain; however, a plateau was not reached in pan-genome size. Since the genome continues to increase and new genes are still being discovered, the pangenome remained open within *L. parabuchneri* species.

**Fig. 4:**
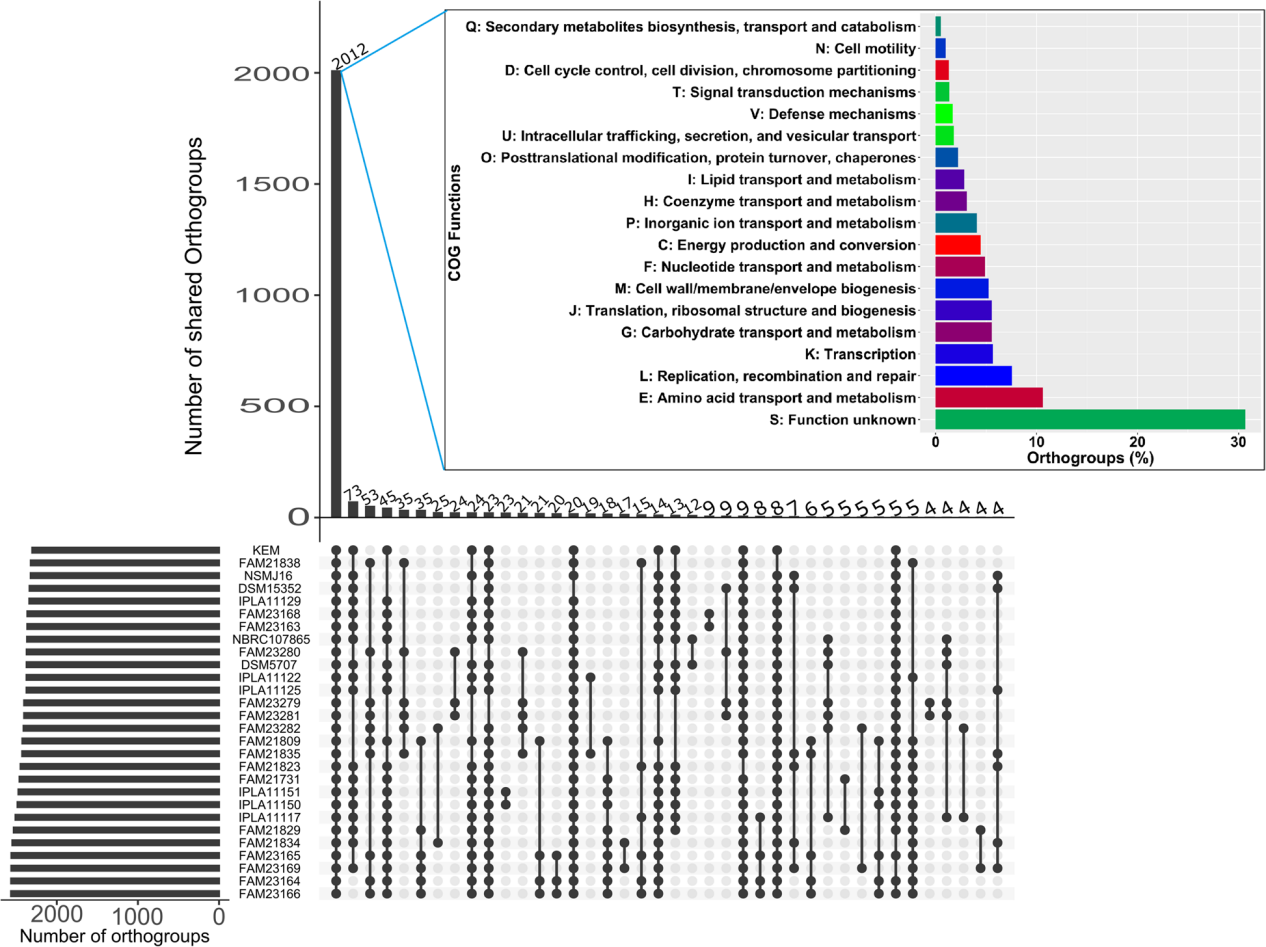
The upSet plot represents the number of orthogroups in each strain and the number of shared orthogroups across the strains with bar charts. UpSetR [30] package in R programming language [27] was utilized to create the figure. The horizontal bar chart in the box shows the functional category of orthogroups based on COG classification using eggNOG-mapper [29]

The UpSet plot represented the shared orthogroup numbers of each strain and shared orthogroup numbers between the strains with a bar chart (Fig. 4). A total of 3451 shared orthogroups were identified by Orthofinder, from which 2012 were identified as core orthogroups, and 1439 were identified as accessory orthogroups (Fig. 4). To elaborate the functions of shared orthgroups across strains, the core orthogroups were classified in functional COG categories using eggNOG-mapper [29]. Interestingly, 29% of the core orthogroups were related to genes with unknown functions. The following frequent functions found were mainly associated with ‘amino acid transport and metabolism’, ‘translation, ribosomal structure and biogenesis’, ‘transcription’, ‘nucleotide transport and metabolism’, ‘carbohydrate transport and metabolism’. The remaining functional COGs were found at lower than 5% frequency (Fig. 4).

**Fig. 5:**
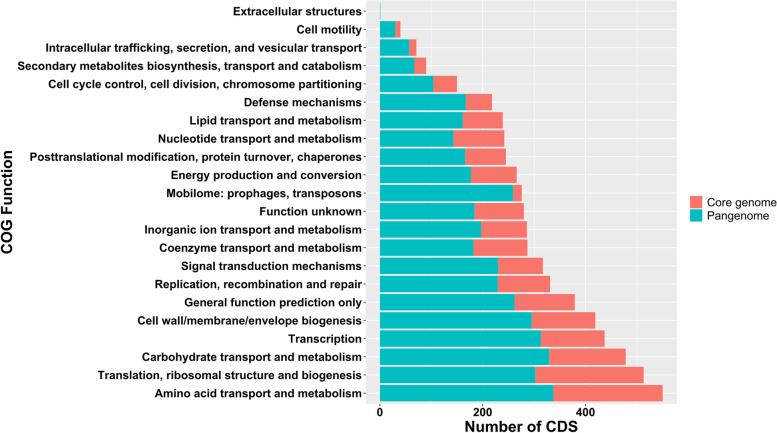
Number of functional COGs in core- (red) and pangenome (light green) of twenty-eight *L. parabuchneri* strains

The pan- and core genomes were annotated using PSI-BLAST followed by COG database [31] and assigned to functional groups (Fig. 5). The two largest core genome categories include CDS with functions associated with translation, ribosomal structure, and biogenesis, as well as amino acid transport and metabolism. The third largest orthogroup, which encodes 7.75% of the total core genome, includes proteins of carbohydrate transport and metabolism. Interestingly, orthogroups encoding ~ 5% of the entire core genome contain proteins of unknown function. Functional core genome categories, including the least number of CDS, belong to ‘cell motility’, ‘intracellular trafficking, secretion, and vesicular transport’, ‘mobilome: prophages, transposons’, and ‘secondary metabolites biosynthesis, transport and catabolism’. Notably, the ‘mobilome: prophages, transposons’ category demonstrated the lowest portion among the number of core genes vs. the number of pan-genes with only 17 CDS in the core genome vs. 259 CDS in the pangenome, implying a great deal of diversity among *L. parabuchneri* strains. Among the 259 mobilomes in pangenome, 179 CDS belonged to transposons or closely associated derivatives.

### Mobile genetic elements

Twenty-eight *L. parabuchneri* genomes were evaluated for the presence of mobilomes such as IS elements, prophages, plasmids, and CRISPR-Cas system. At least two insertion sequences (IS) were identified in all genomes analyzed (Table S3). Among them, FAM23279 and FAM23281 encompassed the highest number of IS elements compared to other *L. parabuchneri* strains studied. By grouping IS families in all *L. parabuchneri* genomes, the highest proportion was found to be the IS30 family (i.e., 67) (Table S3). Genome screening for the presence of prophages and plasmids identified 48 intact prophages and 15 plasmids. Of the 28 *L. parabuchneri* genomes, 22 of them carried at least one intact prophage, and 12 of them harbored at least one plasmid. FAM 23169, FAM21835, and FAM21731 genomes harbored the largest number of intact prophages (i.e., 4). FAM21731 and NSMJ were predicted to encode two and three plasmids, respectively (Table S4). Among the eleven unique plasmids determined, NC_016635.1 was the most abundant plasmid, which comprises 20% of all plasmids identified. The second highest plasmids were NC_002123.1 and NZ_CP018798.1, both of which have been identified four times. The remaining plasmids were identified only once. All the plasmids identified were found in dairy-associated strains except NSMJ16, DSM5707, and NBRC107865, which were isolated from makgeolli, saliva, and unknown source (Table S4). Neither bacteriocin-encoding genes nor antibiotic resistome was found in any *L. parabuchneri* strains analyzed in the present study.

**Fig. 6:**
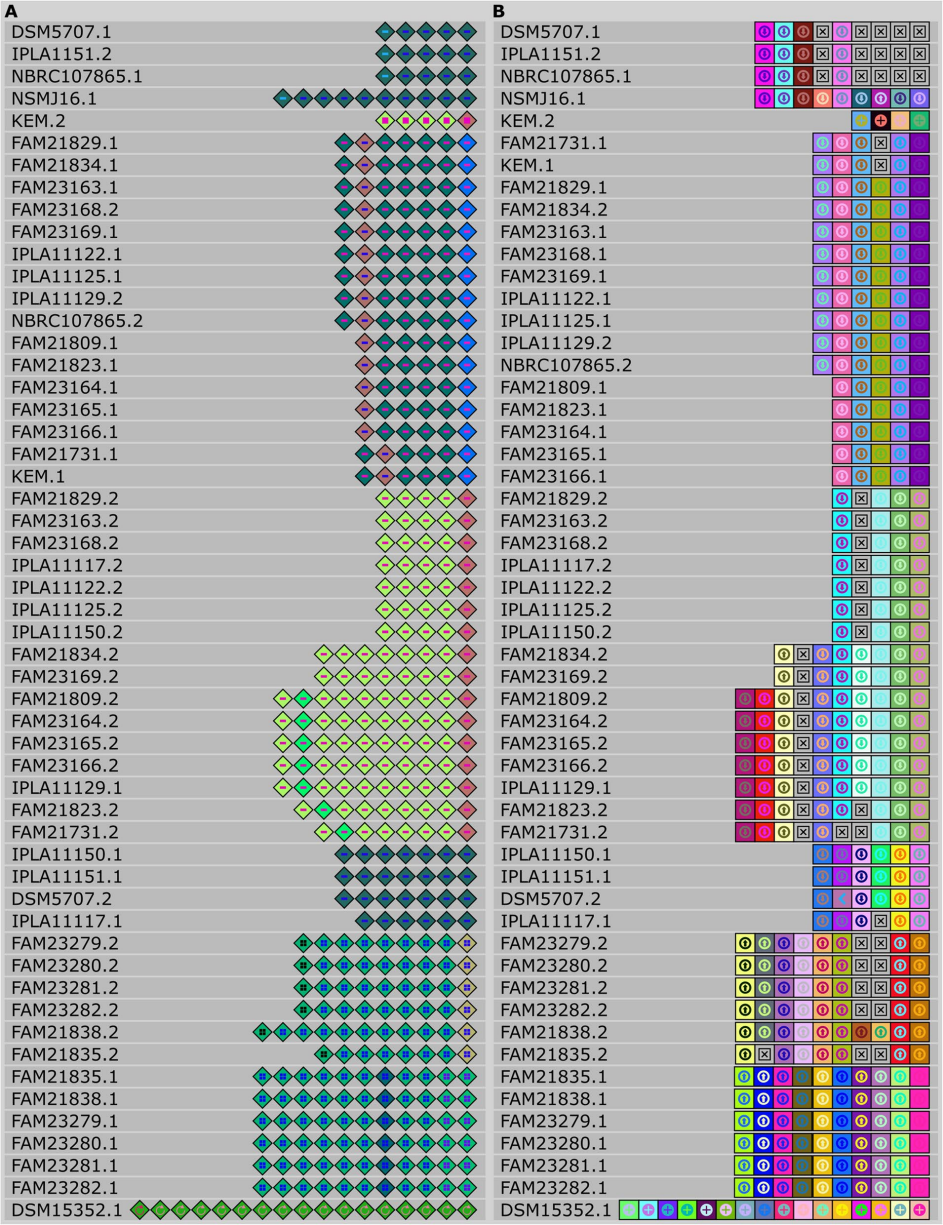
Alignment of repeats (**A**) and spacers (**B**) of each detected CRISPR locus. Each colored diamond represents a unique repeat, and each colored square represents a unique spacer in the CRISPR-Cas system. Grey “x” boxes showed missing spacer

To boost our understanding of CRISPR-Cas systems in-depth, we identified and located repeats (Fig. 6 A) and spacers (Fig. 6B) and successfully assigned them to canonical types and subtypes [32] (Fig. 6). Of twenty-eight strains screened, two different CRISPR-Cas systems were detected which belong to type I-E and II-A canonical subtypes (Table S5). When the subtypes were categorized, a type I-E system was represented in all strains apart from DSM 15352, which only contains the type II-A CRISPR-Cas system. The repeat length ranges from 28 to 35 bp (average ~ 29 bp). DSM5707 has the longest repeat sequence. The alignment of repeats shows ten distinct groups (Table S6). All the strains analyzed carried two CRISPR loci (CRISPR 1 and 2) except DSM15352 and NSMJ16, representing a single CRISPR locus. One strain can appear in two different groups. For example, FAM21809 had two CRISPR loci for its spacer content. CRISPR 1 was part of group 4 whereas CRISPR 2 belonged to group 7 (Table S6).

Identification and alignment of CRISPR spacers also showed ten distinct groups of spacers that share 100% identity in their corresponding group (Table S6). Spacer lengths of the type I-E ranged between 31 and 38 bp (average 32.3 bp), and the length of DSM15352 spacers was 31 bp. DSM5707 had the longest spacer with a length of 38 bp, whereas KEM had the shortest spacer with a length of 31 bp. Although KEM represented the type I-E CRISPR locus, it doesn’t share a significant identity with any type I-E spacers.

### Analysis of Carbohydrate active enzymes

**Fig. 7:**
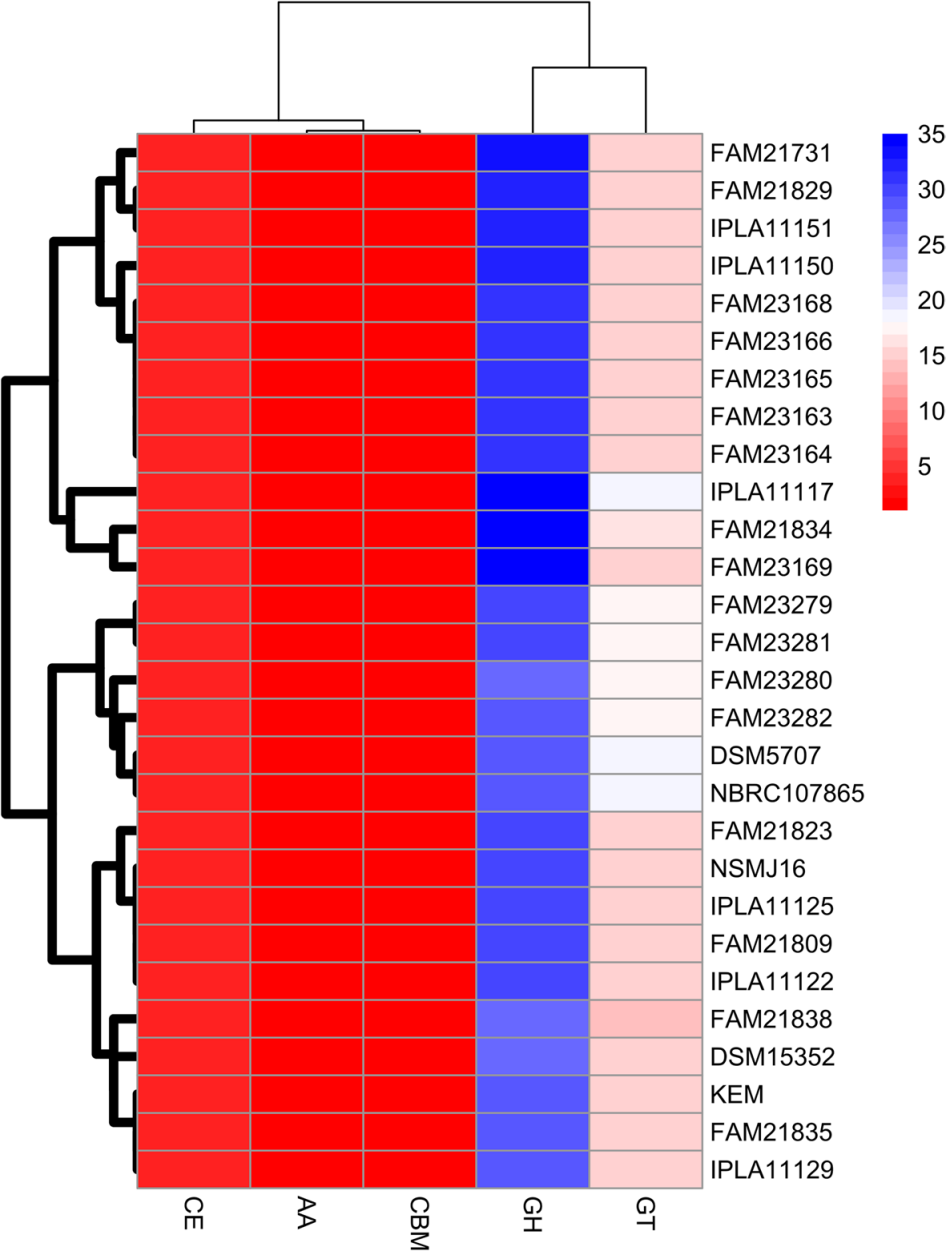
Heatmap of CAZymes distribution and clustering across twenty-eight *L. parabuchneri* genomes. The color gradient from lighter to darker colors represents the abundance of CAZymes found in each genome. GH: Glycoside hydrolase, GT: Glycosyltransferase, CE: Carbohydrate esterase, AA: Auxiliary activity, CBM: Carbohydrate binding module. R programming language (version 4.1.1) [27] was used to draw the heatmap

CAZyme analysis revealed that four distinct clades were formed according to the abundance of genes in each CAZyme family (Fig. 7). IPLA11117, DSM5707, and NBRC107865 had the highest number of GT family enzyme encoding genes. FAM21834, FAM23169, and IPLA11117 were predicted to carry the highest number of GH family CAZyme encoding genes compared to the remaining strains. CBM and CE family CAZymes were carried by all strains at similar abundance. However, AA family CAZymes were missing in 54% of genomes.

### Functional annotation and metabolism

Functional annotation of *L. parabuchneri* strains with KAAS and KEGG Mapper showed that major functional gene categories across all genomes were associated with nucleotide metabolism, translation, and membrane transport in their corresponding functional categories (Fig. S5). The largest standard deviation bars were achieved with membrane transport and translation. The most abundant functional genes related to carbohydrate metabolism were responsible for pyruvate and glycolysis/gluconeogenesis, and amino sugar and nucleotide sugar metabolism. In contrast, ascorbate, aldarate, and inositol phosphate metabolism associated genes were the lowest number in the same category. Major functional genes associated with lipid metabolism consisted of fatty acid biosynthesis, glycerolipid, and glycerophospholipid metabolism genes; however, steroid hormone biosynthesis and fatty acid elongation had the lowest number of genes in the corresponding functional category. Moreover, amino acid metabolism harbors most genes in alanine, aspartate, glutamate, cysteine, and methionine metabolism. Glycine, serine, and threonine metabolism have the largest error bar compared to other functional classes under the amino acid metabolism category (Fig. S5).

We screened twenty-eight genomes with regards to the presence of histidine gene cluster consisting of histidine decarboxylase (*hdcA*), histidine decarboxylase maturation protein (*hdcB*), histidine-tRNA ligase (*hisS*), and histidine/histamine antiporter (*hdcC*). It was found that twenty strains were predicted to carry a complete histidine decarboxylase gene cluster (Table S7). Although all strains had putative *hisS* gene in their genome, the remaining genes in the hdc cluster were missing. Hdc-negative strains were located on the same branch except for NSMJ16 and DSM15352. The twenty histidine-positive strains were segregated into six branches in the phylogenetic tree. The branch members of the reference strain KEM were NSMJ16, FAM21731, FAM21834, and FAM23169. It was interesting to note that hdc-negative DSM15352 was clustered with seven hdc-positive strains in the same branch. Although not a perfect correlation, a relationship was seen between hdc presence and phylogenetic relatedness.

We also screened the genes encoding ADI pathway (i.e., arginine deiminase (*arcA*), ornitihine transcarbamoylase (*arcB*), carbamate kinase (*arcC*), and arginine-ornithine antiporters (*arcD*)) [33] and showed that these four genes were found in each of twenty-eight *L. parabuchneri* strains (Table S7).

Some LAB species have the capability to convert lactate into 1,2-propanediol. The genes required for such a conversion from lactate are lactaldehyde dehydrogenase (*ladH*) and lactaldehyde reductase (*ldr*) [34]. All *L. parabuchneri* genomes analyzed in the present study were predicted to carry both *ladH* and *ldr*.

## Discussion

In the present work, we performed a genome-wide evaluation on the twenty-eight *L. parabuchneri* strains representing milk, cheese, KEM complex, makgeolli, and saliva microbiomes. The genome sizes range between 2.51 Mb to 2.80 Mb, which falls in the range of lactic acid bacteria (i.e., 1.8 to 3.3 Mb). The GC content of *L. parabuchneri* is consistent with low GC LAB. Whole genome analysis identified either single or multiple plasmid sequences in twelve *L. parabuchneri* strains that are inconsistent with no plasmids found in the reference strain of KEM. It was hypothesized that lactic acid bacteria are heavily adapted to their specific ecological niche, which is further supported by the existence of plasmids that could rapidly be gained and transferred at times of swift environmental changes [10].

We also showed similarities or discrepancies in the strain phylogenetic locations across core and whole genome-based sequence alignments. For example, four strains isolated from Tete de Moine cheese (FAM21809, FAM23164, FAM23165, and FAM23166) were closely related in phosphoglucomutase, core genome, and whole genome-based phylogenetic trees (Fig. 1, Fig. S1, Fig. 2). Similarly, six strains isolated from Tilsit, Swiss Alpine Cheese, or milk share the same clade across the core and whole genome-based phylogenetic trees. Noteworthy, the makgeolli isolate of NSMJ16 was the closest genome to KEM among twenty-seven strains according to all phylogenetic trees. Although core genome-based phylogenetic evaluation provided close relatedness of NSMJ16, FAM21731, FAM21834, and FAM23169 against the reference genome, whole genome sequence-based phylogenetic tree revealed NSMJ16, FAM21823, FAM21829, and FAM21731 were the closest strains to KEM. The discrepancies seen between the whole genome vs. core genome-based phylogenetic trees could be attributed to the accessory genome with some contribution of plasmid-encoded genes [35] or inaccurate assemblies (Fig. S1) [36]. For example, Tilsit (FAM21834) and Tete de Moine (FAM23169) isolates were more closely related in the core genome-based tree than whole genome-based alignment (Fig. 1, Fig. S2). The difference seen in both trees could be attributed to unique plasmids found in each strain (Table S4).

Pangenome analysis indicated an open genome, which proposes the functional diversity of *L. parabuchneri*. Pangenome remaining open allows for the continuous acquisition of genetic elements from the external microenvironment and adapt to harsh conditions [37, 38]. We defined a core orthogroup in which all studied genomes were present. *L. parabuchneri* genomes shared 2012 orthogroups which contained genes associated with maintenance that is fundamental to the proliferation and survivability of this species [39].

Detecting 45 intact prophages in ~ 76% of all strains tested and identifying 53 genomic islands based on diverging nucleotide profiles reveal potential horizontal gene transfer hallmark [10]. The proportion of hypothetical/unknown genes in the core genome (~ 41.1%) implies there is still more to discover about *L. parabuchneri*, especially for functional studies. With two-fifths of the identified CDS conserved among all twenty-eight strains analyzed, a remarkable degree of genomic diversity was assigned to the accessory genome. The abundance of prophages, insertion sequences, and genomic islands suggest that mobile elements are likely a crucial genomic trait of *L. parabuchneri.*

*In silico* analysis of the CRISPR-Cas system revealed that all strains screened in the present study encoded a putative CRISPR system. This is higher than lactobacilli overall and bacteria in general, which proposes that *L. parabuchneri* holds a promising potential for unique CRISPR-based tools [40]. Type I forms the most abundant CRISPR-Cas system and could be repurposed as a genetic modification tool upon identification and characterization in their native host [41]. A type I-E CRISPR-Cas system was found in all strains except DSM15352, which harbored type II-A. Across all CRISPR-Cas systems identified, a secondary type I-E loci were detected in all strains except NSMJ16 which was isolated from the Korean traditional alcoholic beverage makgeolli. The smaller number of conserved repeat sequences and unique spacers in CRISPR 2 loci of DSM5707, FAM21829, FAM23163, FAM23168, IPLA11117, IPLA11122, IPLA11125, IPLA11129, IPLA11150, KEM, and NBRC107865 strains might imply CRISPR 2 maintain its functionality post duplication and evolved from CRISPR 1 locus. Similar results were also reported by Nethery et al. (2019) for another fermented food spoilage organism *L. buchneri* [10].

The same CRISPR spacer length and identity were found across IPLA1151, DSM5707, and NBRC107865, and these genomes were found in the same clade (Fig. 1). As expected, cheese isolates of IPLA11150, IPLA11125, and FAM21829, IPLA11117, IPLA11122, FAM23163, and FAM23168 share the same spacer identity and length and conserved repeat regions. Interestingly, the saliva isolate of DSM5707 had identical spacer identity with IPLA11151 and NBRC107865, isolated from cheese and an unknown source, respectively. These three strains shared the first clade in the core genome-based phylogenetic tree. We speculate that DSM5707 might be the transient member of human saliva instead of a permanent member. While NSMJ16 was the closest genome to KEM in phylogenetic trees, no spacer or repeat identity was found across the two strains. Based on these results, we speculate that *L. parabuchneri* strains were remarkably diverse regarding genomic rearrangements and the CRISPR-Cas system. The high spacer diversity in strains isolated from similar origins, such as milk or cheese, proposes each strain’s exposure to various environmental conditions and evolutionary track records [36].

*L. parabuchneri* genome was predicted to encode CAZymes functional in the biosynthesis of carbohydrates and hydrolysis during fermentation. GTs involve in biosynthesis; however, GHs, PLs, CEs, AAs, and CBM participate in degradation. Thus, CAZymes play a key role in carbohydrate metabolism [42]. Of the twelve GT families identified in the *L. parabuchneri* genomes, ~ 54% was represented by GT2 and GT4 families responsible for cellulose synthase, chitin synthase, sucrose synthase, galactosyl transferase, and glucosyl transferase. GH is the main family enzyme that participates in sugar metabolism and plays a key role in the cleavage of carbohydrate glycosidic bonds [42]. Of the twenty GH families determined, ~ 42.6% belonged to GH25, GH73, and GH2. Moreover, GH2 (beta-galactosidase) enzyme encoding gene was available across all *L. parabuchneri* genomes. GH2 functions a key role in lactose metabolism for the growth of the strain in dairy foods [42, 43]. Sugar fermentation capacity is a crucial indicator of a bacterium’s functionality and set the fundamentals for strain selection and cultivation [44]. GH73 or GH25 catalyzes the hydrolysis of the beta-1,4 bond among N-acetyl muramic acid and N-acetyl glucosamine in the cell wall of bacteria; therefore, *L. parabuchneri* might also possess antimicrobial activity [45].

Histamine formation is of particular health concern due to several symptoms caused by ingestion of histamine [46] catalyzed by the histidine decarboxylase gene cluster (*hdcA*, *hdcB*, *hisS*, and *hdcC*) [8, 47]. *L. parabuchneri* strains were repeatedly isolated from cheese, showing an increased histamine content [47]. We screened the hdc gene cluster responsible for histidine to histamine conversion in twenty-eight strains and found that twenty-two strains had putative hdc gene cluster (Table S7). In a previous study by Wüthrich et al. (2017), twelve hdc-positive *L. parabuchneri* strains were primarily isolated from cheese [8]. It was proposed that *L. parabuchneri* acquired the hdc gene cluster through a horizontal gene transfer [8]. Here we feed the pipeline of putative hdc-positive *L. parabuchneri* strains that were not only isolated from cheese but also originated from saliva, KEM, and unknown sources. This implies that hdc-positive *L. parabuchneri* strains are more prevalent than initially thought and exceed the environments beyond the cheese microbiome. It was demonstrated that the hdc gene cluster could be utilized for energy production and pH regulation [48]. Moreover, carrier-mediated transport produces a proton motive force [49]. We would anticipate that the hdc gene cluster confers a competitive advantage over *L. parabuchneri* strains in the cheese microenvironment. Although this might be seen as a beneficial trait during the acidification and ripening of cheese making, the end product, histamine, causes intolerance reactions in consumers [8].

LAB utilizes the arginine deiminase pathway for converting arginine into ornithine by citrulline while producing ATP and ammonia. Production of ammonia elevates the pH so that bacteria are protected against conditions of an acid-stress [50]. We show that all twenty-eight *L. parabuchneri* genomes analyzed in the present study carried putative *arcA*, *arcB*, *arcC*, and *arcD*, which catalyze the ADI pathway. In carbohydrate-deficient but amino acid-rich conditions such as ripening cheese, the ADI pathway is an important avenue to produce ATP for bacterial proliferation [33]. It was shown that *L. parabuchneri* ADI metabolism is a helpful trait during cheese ripening where pH is reduced due to acidification, and bacteria that are not capable of adapting to this low pH would be outgrown or neutralized [33]. Therefore, *L. parabuchneri* can increase its biomass in high salt in moisture and acidic conditions of cheese ripening. The ammonia produced in the ADI pathway also enhances the proteolysis in the cheese ripening [13, 14]. Moreover, CO_2_ released contributes to the increase in the quantity and size of holes in semi-hard cheese [13]. However, the outgrowth of *L. parabuchneri* in long-ripened cheese varieties could be associated with the unwanted gas formation and splits that can cause downgrading of cheese and cutting losses resulting in severe economic losses to cheese manufacturers [13, 51–54].

*L. parabuchneri* genomes present metabolic pathways for the conversion of lactate to 1,2-propanediol perhaps to cope with acid stress in the microenvironment and to produce ATP. All *L. parabuchneri* genomes studied in the present work were predicted to carry genes required for lactate to 1,2-propanediol conversion. First, lactate is transformed to L-lactaldehyde by lactaldehyde dehydrogenase enzyme then L-lactaldehyde is reduced to 1,2-propanediol through lactaldehyde reductase. This degradation of two moles of lactate generates one mole of ATP [9]. It was reported that the conversion of lactate to 1,2-propanediol strictly depends on the environment’s pH as low pH values induce the degradation process [9]. This could be a stress response metabolism *L. parabuchneri* developed to protect against surrounding acidic conditions during cheese make and ripening conditions. Conversion of lactate by *L. parabuchneri* was reported to be a minor factor in the CO_2_ production [13].

Cheese defects of burning taste, crack formation, and histamine outbreaks due to *L. parabuchneri* contamination and outgrowth are significant industrial concerns and health risks [14, 55]. The putative intact prophages found in *L. parabuchneri* should be explored in vivo to reduce the incidence of unwanted product quality and health-associated symptoms caused by this species. In particular, the deliberate induction strategies against putative prophages for controlling contamination and proliferation of this organism should require further attention. Bioprotection agents such as bacteriocins or bioprotective adjunct cultures could also be explored to limit this species’ population and cheese defects caused by *L. parabuchneri*.

## Conclusion

The goal of the present work was to (i) improve core knowledge on *L. parabuchneri*, a lactobacilli species primarily associated with unwanted gas formation, off-flavor, and elevated histamine formation in ripening cheese, (ii) identify novel tools and strategies based on genomic analysis to combat with this organism causing both economic loss and health concerns worldwide. The phylogenetic analysis based on the core genome sequence alignment revealed four distinct clades. Saliva, KEM, and makgeolli form the same clade, with dairy-originated strains showing evidence of diversity. No pronounced differences seen in carbohydrate-active enzymes according to the origins of each strain imply a free-living lifestyle. The frequency of type I-E CRISPR-Cas system in all strains but one is consistent with the high occurrence of type I-E across all types. The abundance of IS elements, genomic islands, and intact prophages found in the majority of strains revealed the plasticity of the genome. In particular, prophages could be further studied in vivo to determine their activity which could evolve into prophage mediated lysis strategy, thus potentially helping to reduce the contamination and growth of this unwanted microbe in fermented dairy foods.

## Methods

Whole genome sequences of thirty *L. parabuchneri* genomes were downloaded from NCBI GenBank, followed by a BUSCO analysis to inspect the completeness of the genome assemblies using the lactobacillales_odb10 lineage dataset [56, 57]. The genome assemblies showing greater than 95% completeness were annotated using RAST [58–60] and Prokka (version 1.14.6) [61]. The two genomes (VRA_07sq_f and FAM23167) showing < 95% BUSCOs were discarded.

RAST annotation outputs were analyzed to predict putative cas genes [10]. The core- and pangenome analysis was conducted by annotating genomes first with Prokka [61] with the following arguments: --kingdom Bacteria --compliant. Then, output files from Prokka were sent to Roary (version 3.13.0) [23] using arguments: -e -n -v -r to carry out the analysis. Pan- and core genes were assigned a functional COG using PSI-BLAST [62] with the following flags: -show_gis -outfmt 7 -num_descriptions 1000 -num_alignments 1000 -dbsize 100,000,000 -comp_ based_stats T -seg yes [10]. The COG database [31] is publicly available for download with the following link: https://www.ncbi.nlm.nih.gov/COG/. Core and Pan COGs were visualized using R [27] and ggplot2 [28].

A global phylogenetic analysis was conducted based on the phosphoglucomutase gene [63], core genes, and the whole genome. After extracting the phosphoglucomutase gene sequences and core genome from Prokka, nucleotide sequences were aligned using Clustal Omega [64]. A phylogenetic tree was generated using the iTOL web tool [65]. The whole genome-based phylogenetic tree was created using TYGS online tool [25]. Genome-wide alignment of each genome was performed using the BLAST Ring Image Generator (BRIG) tool [66] against the reference strain KEM. A ring for each genome was included in addition to GC content and GC skew. The BLASTn [62] was utilized with upper and lower identity thresholds of 90% and 70%, respectively, with a ring size of 10. Shared orthogroups across the genomes were identified using OrthoFinder [67] with default settings, and an UpSet plot was constructed using R [27] with the UpSetR package [30]. Core orthogroups shared between *L. parabuchneri* strains were annotated to clusters of orthologous groups (COG) categories using eggNOG-mapper [29].

CAZy database (v10) in dbCAN server (https://bcb.unl.edu/dbCAN2/index.php) [68] and HMMER (version 3.3.2) [69] were utilized to identify Carbohydrate active enzyme (CAZyme) related genes according to suggested protocol dbCAN. Results of the CAZYme analysis were filtered with the recommended threshold of minimum 0.35 coverage and E-value 1e-15 according to Oliviera et al. (2022) [39]. Then, *L. parabuchneri* strains were classified based on the number of CAZYmes they harbored. KEGG Automatic Annotation Server (KAAS) was utilized for functional annotation of *L. parabuchneri* strains with the assignment method of the bi-directional best hit (BBH) method [70]. Results from KAAS were analyzed to identify the number of genes associated with metabolic pathways and functional classes by utilizing the KEGG Mapper web tool [71, 72].

Identification, alignment, and visualization of CRISPR elements, such as repeats and spacers, were conducted with the CRISPRviz tool [73]. Classification of CRISPR-Cas loci was determined according to Koonin et al. (2017) with flanking cas genes and their corresponding annotations [32]. CRISPRCasFinder [74] was also utilized to confirm CRISPR types. Genomic islands were determined using GIPSy [75] with default settings. Identification of plasmids in genomes of *L. parabuchneri* was performed with PLSDB (version 2021_06_23_v2) using default settings [76, 77]. Phage Search Tool Enhanced Release (PHASTER) was utilized to identify the prophages [78]. Insertion sequences were identified by ISfinder tool [79]. To identify potential bacteriocins and bacteriocin-expressing regions BAGEL4 web tool was utilized [80]. Screening of antibiotic resistance genes was performed by CARD web tool [81].

## Supplementary Information

**Additional file1.**

## Data Availability

Genomes analyzed in the present study are available in NCBI with the following accession numbers: DSM 15,352 (GCA_001437335.1), DSM 5707 (GCA_001435315.1), FAM 23,169 (GCA_005864155.1), FAM21731 (GCA_001922025.1), FAM21809 (GCA_002095795.1), FAM21823 (GCA_002095615.1), FAM21829 (GCA_002095645.1), FAM21834 (GCA_002095655.1), FAM21835 (GCA_002095755.1), FAM21838 (GCA_002095635.1), FAM23163 (GCA_002095835.1), FAM23164 (GCA_002095845.1), FAM23165 (GCA_002095695.1), FAM23166 (GCA_002095725.1), FAM23167 (GCA_002095815.1), FAM23168 (GCA_002095715.1), FAM23169 (GCA_002095825.1), FAM23279 (GCA_002095895.1), FAM23280 (GCA_002095905.1), FAM23281 (GCA_002095915.1), FAM23282 (GCA_002095765.1), IPLA 11,117 (GCA_001687155.1), IPLA 11,122 (GCA_001677035.1), IPLA 11,150 (GCA_001687145.1), IPLA11125 (GCA_019266025.1), IPLA11129 (GCA_019266005.1), IPLA11151 (GCA_019265985.1), KEM (GCA_014879295.1), NBRC 107,865 (GCA_001591885.1), NSMJ16 (GCA_014905035.1), and VRA_07sq_f (GCA_009683085.1). All plasmids discovered in the present study are available in NCBI GenBank with the following accession numbers: FAM21731 (NZ_CP018798.1), FAM21731 (NZ_CP018797.1), FAM23169 (NC_016635.1), NSMJ16 (NZ_CP050496.1), NSMJ16 (NZ_CP050495.1), NSMJ16 (NZ_CP050494.1), FAM21823 (NZ_CP017265.1), FAM21829 (NZ_CP018798.1), IPLA11150 (NC_016635.1), IPLA11151 (NC_016635.1), NBRC107865 (NC_002123.1), DSM5707 (NC_002123.1), FAM21834 (NZ_LM651913.1), DSM15352 (NZ_CP047122.1), IPLA11129 (NZ_CP065817.1).
