# Supplementary material for: Comparative Genomics of Lentilactobacillus parabuchneri isolated from dairy, KEM complex, Makgeolli, and Saliva Microbiomes
Source: BMC Genomics. 2022 Dec 5;23:803. doi: 10.1186/s12864-022-09053-y (PMC9724434; doi:10.1186/s12864-022-09053-y)
Supplement: Supplementary file 1 — Additional file1. [file 12864_2022_9053_MOESM1_ESM.docx]

**Supplementary Figures and Tables**

# Comparative genomics of *Lentilactobacillus parabuchneri* isolated from dairy, KEM complex, Makgeolli, and saliva microbiomes

Ismail Gumustop^1^, Fatih Ortakci^1^*

^1^BioEngineering Department, Faculty of Life and Natural Sciences, Abdullah Gul University, Kayseri, TR

*Corresponding author: [fatih.ortakci@agu.edu.tr](mailto:fatih.ortakci@agu.edu.tr)


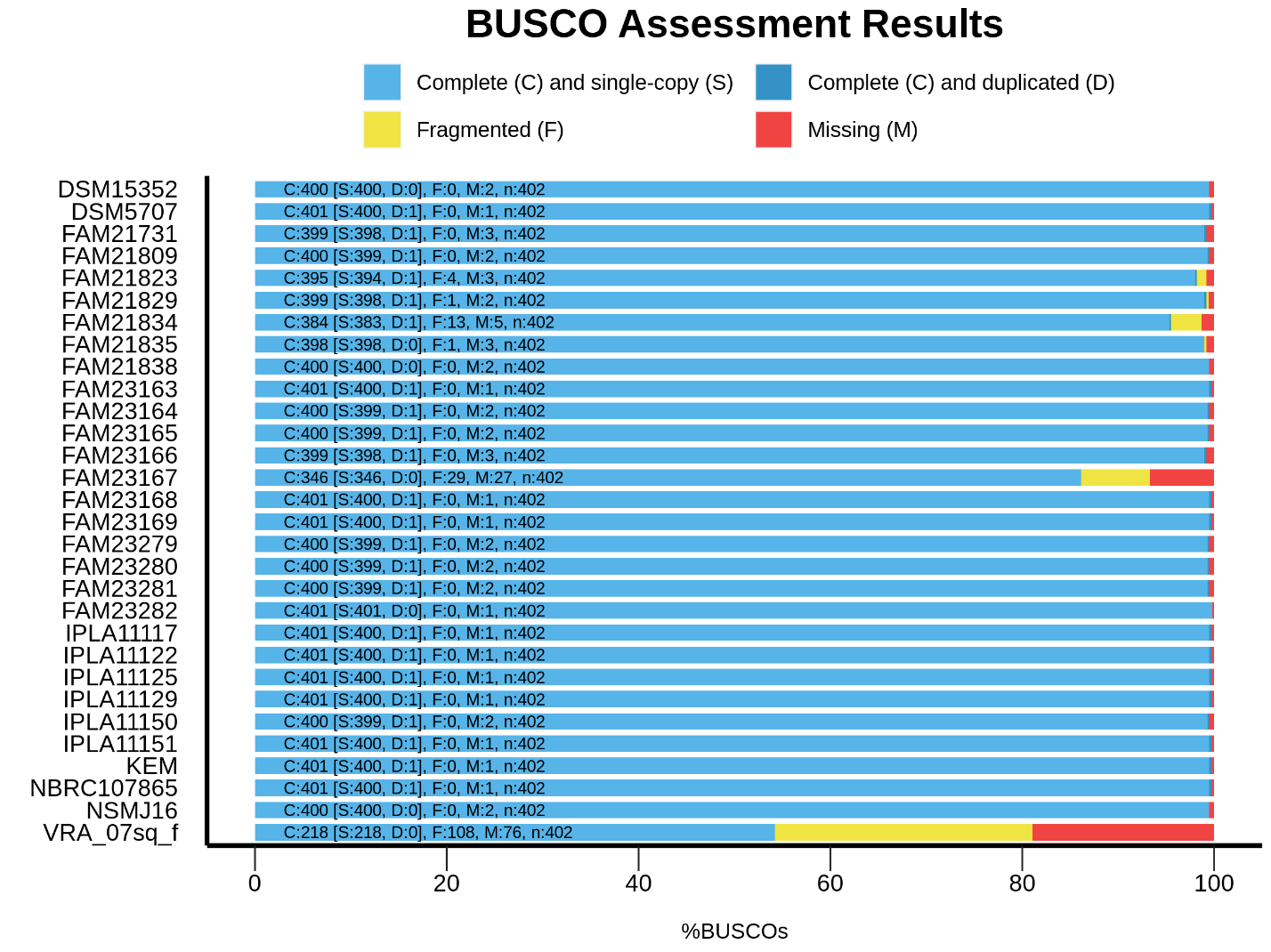


**Figure S1.** BUSCO assessment results of 30 *L. parabuchneri* genomes assemblies.


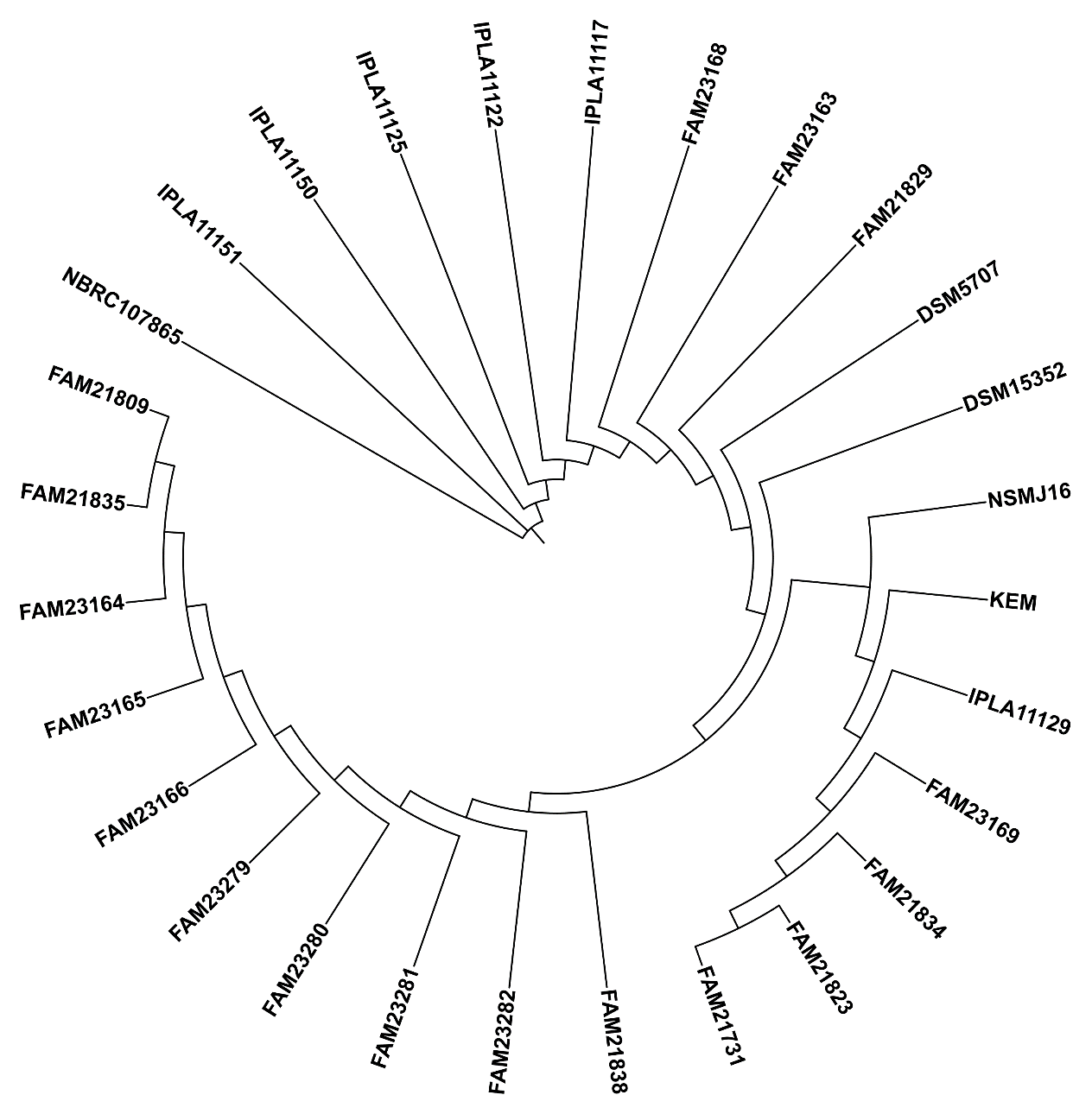


**Figure S2.** Phylogenetic tree of multiple sequence comparison of phosphoglucomutase genes.


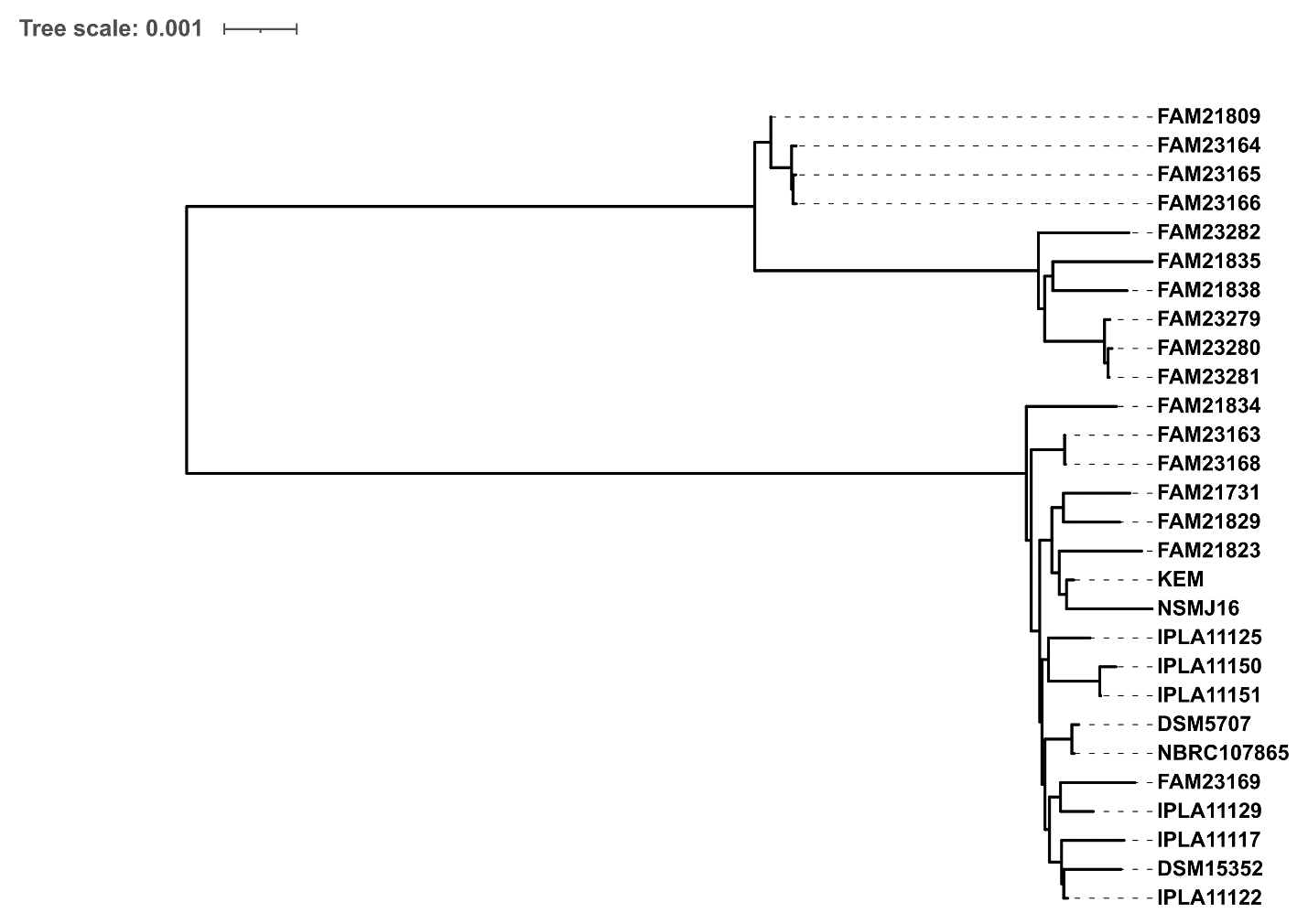


**Figure S3.** Whole genome based phylogenetic tree of twenty-eight *L. parabuchneri*.


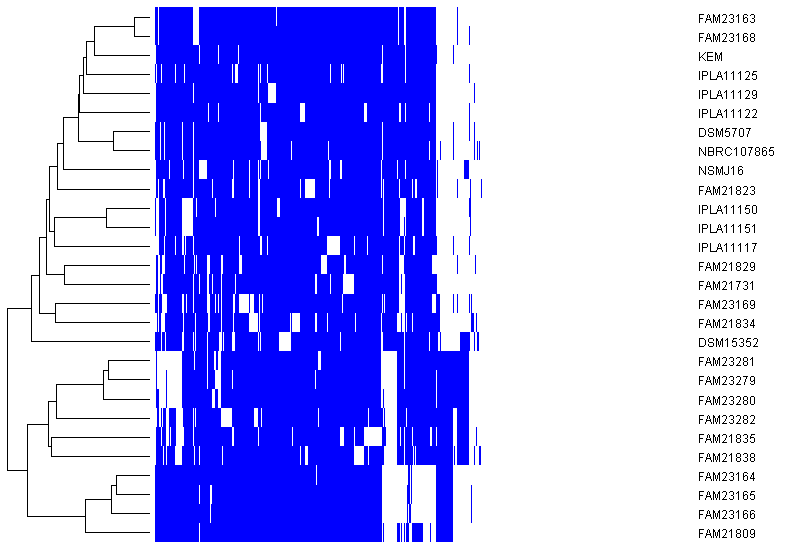


**Figure S4.** Representation of gene cluster matrix based on gene absence-presence. R programming language [23] was used to create the cluster matrix.


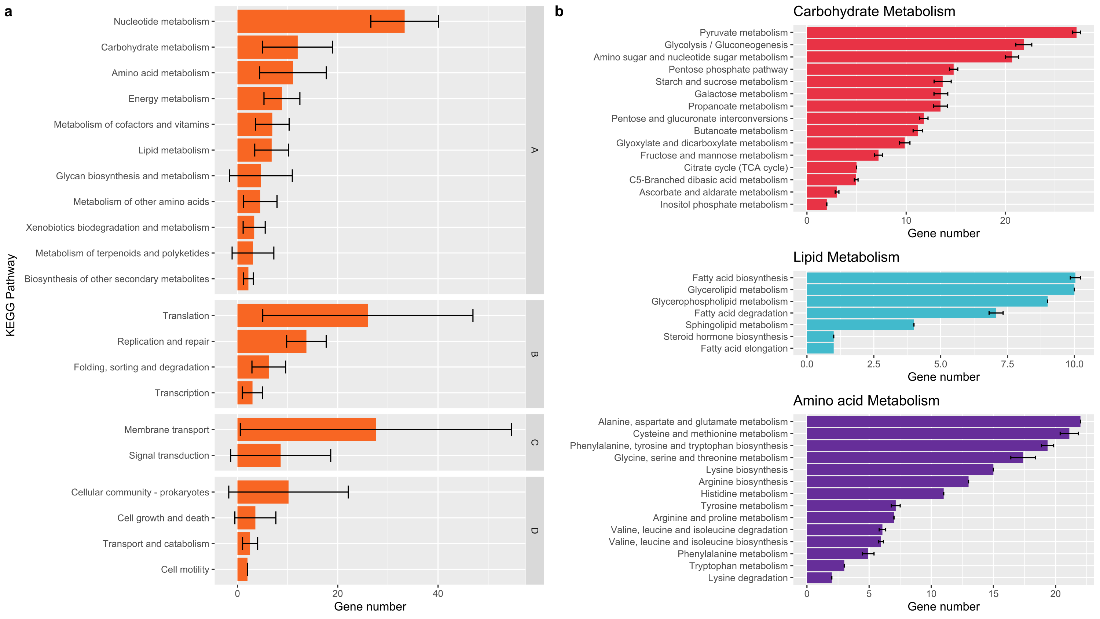


**Figure S5.** KAAS-KEGG functional annotations a) detailed representation of functional classes belonging to five main functional categories b) subcategories of carbohydrate, lipid, and amino acid metabolism. Functional categories: **A** Metabolism, **B** Genetic Information Processing, **C** Environmental Information Processing, **D** Cellular Processes

**Table S1**. Genomic islands on genomes of L. parabuchneri and their locations on their corresponding genome.

| Putative genomic island | Start | End | Prediction score |
| --- | --- | --- | --- |
| Genomic Island 1 | 4405 | 10840 | Normal |
| Genomic Island 2 | 51319 | 72607 | Normal |
| Genomic Island 3 | 51319 | 72607 | Normal |
| Genomic Island 4 | 51319 | 72607 | Normal |
| Genomic Island 5 | 52393 | 59118 | Normal |
| Genomic Island 6 | 106707 | 115819 | Normal |
| Genomic Island 7 | 180284 | 225899 | Normal |
| Genomic Island 8 | 234962 | 241386 | Normal |
| Genomic Island 9 | 247694 | 254127 | Normal |
| Genomic Island 10 | 270192 | 277310 | Normal |
| Genomic Island 11 | 314028 | 334027 | Normal |
| Genomic Island 12 | 520365 | 531540 | Normal |
| Genomic Island 13 | 520365 | 531540 | Normal |
| Genomic Island 14 | 544365 | 560936 | Normal |
| Genomic Island 15 | 665814 | 674807 | Normal |
| Genomic Island 16 | 743674 | 754995 | Normal |
| Genomic Island 17 | 743674 | 754995 | Normal |
| Genomic Island 18 | 839437 | 863027 | Normal |
| Genomic Island 19 | 839437 | 863027 | Normal |
| Genomic Island 20 | 943411 | 985419 | Strong |
| Genomic Island 21 | 1060057 | 1081305 | Normal |
| Genomic Island 22 | 1060057 | 1081305 | Normal |
| Genomic Island 23 | 1078129 | 1085261 | Normal |
| Genomic Island 24 | 1116703 | 1126957 | Normal |
| Genomic Island 25 | 1121539 | 1143288 | Normal |
| Genomic Island 26 | 1179592 | 1195129 | Normal |
| Genomic Island 27 | 1182721 | 1217521 | Normal |
| Genomic Island 28 | 1182721 | 1217521 | Normal |
| Genomic Island 29 | 1443988 | 1467975 | Normal |
| Genomic Island 30 | 1459026 | 1476971 | Strong |
| Genomic Island 31 | 1508516 | 1529971 | Normal |
| Genomic Island 32 | 1519422 | 1570906 | Normal |
| Genomic Island 33 | 1612462 | 1627490 | Normal |
| Genomic Island 34 | 1698116 | 1710094 | Normal |
| Genomic Island 35 | 1773754 | 1781772 | Normal |
| Genomic Island 36 | 1773754 | 1781772 | Normal |
| Genomic Island 37 | 1779539 | 1788352 | Normal |
| Genomic Island 38 | 1789278 | 1839690 | Normal |
| Genomic Island 39 | 1817095 | 1825055 | Normal |
| Genomic Island 40 | 1862528 | 1873519 | Normal |
| Genomic Island 41 | 1882679 | 1910293 | Normal |
| Genomic Island 42 | 1918009 | 1944783 | Normal |
| Genomic Island 43 | 1930892 | 1939476 | Normal |
| Genomic Island 44 | 1969379 | 1976499 | Normal |
| Genomic Island 45 | 1991737 | 2000534 | Normal |
| Genomic Island 46 | 2099245 | 2123814 | Normal |
| Genomic Island 47 | 2122157 | 2168324 | Normal |
| Genomic Island 48 | 2125820 | 2171552 | Normal |
| Genomic Island 49 | 2258512 | 2279736 | Normal |
| Genomic Island 50 | 2324363 | 2332527 | Strong |
| Genomic Island 51 | 2375117 | 2383294 | Normal |
| Genomic Island 52 | 2417915 | 2435352 | Strong |
| Genomic Island 53 | 2417915 | 2435352 | Strong |

**Table S2.** Putative intact prophages predicted in twenty *L. parabuchneri* strains using PHASTER

| **Strain** | **Region** | **Region Length** | **Completeness** | **Score** | **# Total Proteins** | **Start** | **End** | **Most Common Phage** | **GC %** |
| --- | --- | --- | --- | --- | --- | --- | --- | --- | --- |
| FAM 23169 | 2 | 71.5Kb | intact | 140 | 71 | *725253* | *796826* | PHAGE_Lactob_LBR48_NC_027990(22) | 42.43 |
| FAM 23169 | 4 | 37.9Kb | intact | 140 | 50 | *2240585* | *2278539* | PHAGE_Lactob_Lj771_NC_010179(20) | 40.45 |
| FAM 23169 | 8 | 18.5Kb | intact | 140 | 29 | *2761532* | *2780092* | PHAGE_Staphy_phiPV83_NC_002486(2) | 40.50 |
| FAM 23169 | 9 | 12.9Kb | intact | 140 | 28 | *2780739* | *2793640* | PHAGE_Paenib_Tripp_NC_028930(3) | 41.71 |
| FAM21731 | 1 | 45.1Kb | intact | 130 | 57 | *1961360* | *2006521* | PHAGE_Lactob_T25_NC_048625(11) | 37.42 |
| FAM21731 | 2 | 53.3Kb | intact | 150 | 78 | *2197976* | *2251307* | PHAGE_Lactob_LBR48_NC_027990(21) | 41.16 |
| FAM21731 | 4 | 34Kb | intact | 150 | 47 | *2603577* | *2637665* | PHAGE_Salmon_SJ46_NC_031129(2) | 39.86 |
| FAM21731 | 5 | 15.5Kb | intact | 120 | 15 | *2693460* | *2708963* | PHAGE_Lactob_phiAT3_NC_005893(2) | 42.34 |
| FAM21809 | 1 | 45.4Kb | intact | 150 | 62 | *52391* | *97870* | PHAGE_Lactob_jlb1_NC_024206(20) | 40.05 |
| FAM21809 | 2 | 42.4Kb | intact | 100 | 53 | *1943803* | *1986276* | PHAGE_Lactob_T25_NC_048625(11) | 37.61 |
| FAM21809 | 3 | 49.3Kb | intact | 150 | 80 | *2174794* | *2224159* | PHAGE_Lactob_LBR48_NC_027990(21) | 41.48 |
| FAM21823 | 1 | 43.9Kb | intact | 110 | 54 | *1983183* | *2027180* | PHAGE_Lactob_T25_NC_048625(11) | 37.62 |
| FAM21829 | 2 | 41.5Kb | intact | 150 | 60 | *1854856* | *1896405* | PHAGE_Lactob_jlb1_NC_024206(20) | 39.74 |
| FAM21829 | 3 | 50.2Kb | intact | 150 | 77 | *2082327* | *2132593* | PHAGE_Lactob_LBR48_NC_027990(22) | 41.40 |
| FAM21834 | 4 | 50.7Kb | intact | 150 | 78 | *2145133* | *2195923* | PHAGE_Lactob_LBR48_NC_027990(22) | 41.71 |
| FAM21834 | 8 | 14.9Kb | intact | 150 | 21 | *2743735* | *2758682* | PHAGE_Paenib_Tripp_NC_028930(3) | 41.97 |
| FAM21835 | 1 | 41.7Kb | intact | 120 | 50 | *1833437* | *1875181* | PHAGE_Lactob_T25_NC_048625(11) | 37.60 |
| FAM21835 | 5 | 12.4Kb | intact | 100 | 18 | *2550268* | *2562737* | PHAGE_Salmon_SJ46_NC_031129(2) | 40.95 |
| FAM21835 | 6 | 17.6Kb | intact | 150 | 21 | *2610067* | *2627687* | PHAGE_Paenib_Xenia_NC_028837(2) | 41.51 |
| FAM21835 | 7 | 14.6Kb | intact | 140 | 21 | *2649194* | *2663802* | PHAGE_Bacter_Diva_NC_028788(2) | 42.28 |
| FAM23163 | 1 | 42.6Kb | intact | 100 | 48 | *1820853* | *1863540* | PHAGE_Lactob_T25_NC_048625(11) | 37.54 |
| FAM23164 | 3 | 41.7Kb | intact | 100 | 50 | *1928032* | *1969749* | PHAGE_Lactob_T25_NC_048625(11) | 37.64 |
| FAM23164 | 4 | 84.3Kb | intact | 150 | 133 | *2158582* | *2242932* | PHAGE_Lactob_LBR48_NC_027990(23) | 41.06 |
| FAM23165 | 1 | 45.2Kb | intact | 150 | 63 | *50050* | *95315* | PHAGE_Lactob_Lj771_NC_010179(20) | 39.97 |
| FAM23165 | 2 | 41.7Kb | intact | 100 | 52 | *1957432* | *1999149* | PHAGE_Lactob_T25_NC_048625(11) | 37.64 |
| FAM23165 | 3 | 48Kb | intact | 150 | 74 | *2187982* | *2236017* | PHAGE_Lactob_LBR48_NC_027990(21) | 41.47 |
| FAM23166 | 2 | 41.7Kb | intact | 100 | 49 | *1905500* | *1947217* | PHAGE_Lactob_T25_NC_048625(11) | 37.64 |
| FAM23166 | 3 | 53.7Kb | intact | 140 | 82 | *2042780* | *2096564* | PHAGE_Lactob_Lj771_NC_010179(20) | 40.29 |
| FAM23166 | 4 | 49.3Kb | intact | 150 | 80 | *2184747* | *2234112* | PHAGE_Lactob_LBR48_NC_027990(21) | 41.48 |
| FAM23168 | 1 | 42.6Kb | intact | 100 | 49 | *1820677* | *1863364* | PHAGE_Lactob_T25_NC_048625(11) | 37.54 |
| FAM23169 | 1 | 37.4Kb | intact | 100 | 50 | *1889912* | *1927354* | PHAGE_Lactob_T25_NC_048625(7) | 37.51 |
| FAM23169 | 2 | 84.2Kb | intact | 150 | 124 | *2122611* | *2206826* | PHAGE_Lactob_LBR48_NC_027990(24) | 41.05 |
| FAM23169 | 5 | 27.6Kb | intact | 150 | 44 | *2768655* | *2796334* | PHAGE_Paenib_Tripp_NC_028930(3) | 41.12 |
| FAM23281 | 2 | 42.9Kb | intact | 100 | 58 | *1876982* | *1919950* | PHAGE_Lactob_Sha1_NC_019489(7) | 37.76 |
| FAM23281 | 5 | 7.9Kb | intact | 150 | 17 | *2607442* | *2615373* | PHAGE_Paenib_Tripp_NC_028930(2) | 42.32 |
| FAM23282 | 2 | 46.7Kb | intact | 140 | 63 | *1467315* | *1514109* | PHAGE_Oenoco_phiS13_NC_023560(13) | 39.32 |
| FAM23282 | 3 | 44.2Kb | intact | 100 | 54 | *1770475* | *1814697* | PHAGE_Lactob_T25_NC_048625(12) | 37.74 |
| IPLA 11117 | 5 | 53.7Kb | intact | 150 | 44 | *1491600* | *1545331* | PHAGE_Lactob_LfeSau_NC_029068(5) | 38.96 |
| IPLA 11122 | 1 | 57.6Kb | intact | 150 | 76 | *103* | *57799* | PHAGE_Lactob_LBR48_NC_027990(10) | 40.01 |
| IPLA 11122 | 2 | 41Kb | intact | 100 | 50 | *242488* | *283499* | PHAGE_Lactob_T25_NC_048625(11) | 37.99 |
| IPLA 11150 | 2 | 51.4Kb | intact | 140 | 74 | *218155* | *269641* | PHAGE_Lactob_LBR48_NC_027990(24) | 41.74 |
| IPLA11125 | 1 | 42.9Kb | intact | 120 | 55 | *225697* | *268646* | PHAGE_Lactob_T25_NC_048625(11) | 37.53 |
| IPLA11129 | 1 | 37.5Kb | intact | 100 | 43 | *239156* | *276748* | PHAGE_Lactob_T25_NC_048625(11) | 37.58 |
| IPLA11151 | 1 | 62.2Kb | intact | 140 | 86 | *184869* | *247068* | PHAGE_Lactob_LBR48_NC_027990(24) | 41.60 |
| KEM | 2 | 43.1Kb | intact | 100 | 53 | *1958716* | *2001847* | PHAGE_Lactob_Sha1_NC_019489(8) | 37.78 |

**Table S3.** Putative insertion sequence (IS) elements of 28 *L. parabuchneri* strains

| **Strain** | **Sequences producing significant alignments** | **IS Family** | **Group** | **Origin** | **Score (bits)** | **E. value** |
| --- | --- | --- | --- | --- | --- | --- |
| DSM 5707 | IS1165 | ISL3 |  | Leuconostoc mesenteroides | 2960 | 0 |
| DSM 5707 | IS1310 | IS256 |  | Enterococcus hirae | 2173 | 0 |
| DSM 5707 | IS153 | IS3 | IS3 | Lactobacillus sanfranciscensis | 823 | 0 |
| FAM21731 | IS1310 | IS256 |  | Enterococcus hirae | 2339 | 0 |
| FAM21731 | ISLpl1 | IS30 |  | Lactobacillus plantarum | 1988 | 0 |
| FAM21731 | ISPp1 | IS30 |  | Pediococcus pentosaceus | 1869 | 0 |
| FAM21731 | IS153 | IS3 | IS3 | Lactobacillus sanfranciscensis | 1126 | 0 |
| FAM21809 | ISLpl1 | IS30 |  | Lactobacillus plantarum | 2004 | 0 |
| FAM21809 | ISPp1 | IS30 |  | Pediococcus pentosaceus | 1901 | 0 |
| FAM21823 | IS1310 | IS256 |  | Enterococcus hirae | 2068 | 0 |
| FAM21823 | IS1165 | ISL3 |  | Leuconostoc mesenteroides | 1737 | 0 |
| FAM21823 | ISLpl1 | IS30 |  | Lactobacillus plantarum | 1600 | 0 |
| FAM21823 | ISPp1 | IS30 |  | Pediococcus pentosaceus | 1544 | 0 |
| FAM21823 | IS153 | IS3 | IS3 | Lactobacillus sanfranciscensis | 973 | 0 |
| FAM21829 | ISLsa1 | IS30 |  | Lactobacillus sakei | 2040 | 0 |
| FAM21829 | ISLpl1 | IS30 |  | Lactobacillus plantarum | 1703 | 0 |
| FAM21829 | ISPp1 | IS30 |  | Pediococcus pentosaceus | 1616 | 0 |
| FAM21829 | IS1310 | IS256 |  | Enterococcus hirae | 1380 | 0 |
| FAM21829 | IS153 | IS3 | IS3 | Lactobacillus sanfranciscensis | 1126 | 0 |
| FAM21834 | IS1165 | ISL3 |  | Leuconostoc mesenteroides | 2896 | 0 |
| FAM21834 | IS1310 | IS256 |  | Enterococcus hirae | 2212 | 0 |
| FAM21834 | ISLpl1 | IS30 |  | Lactobacillus plantarum | 2004 | 0 |
| FAM21834 | ISPp1 | IS30 |  | Pediococcus pentosaceus | 1901 | 0 |
| FAM21835 | IS1165 | ISL3 |  | Leuconostoc mesenteroides | 2896 | 0 |
| FAM21835 | ISLpl2 | IS3 | IS150 | Lactobacillus plantarum | 2422 | 0 |
| FAM21835 | IS1310 | IS256 |  | Enterococcus hirae | 2347 | 0 |
| FAM21835 | ISLdl3 | IS30 |  | Lactobacillus delbrueckii | 2284 | 0 |
| FAM21835 | ISLhe30 | IS30 |  | Lactobacillus helveticus | 1635 | 0 |
| FAM21835 | IS153 | IS3 | IS3 | Lactobacillus sanfranciscensis | 910 | 0 |
| FAM21835 | ISLpl1 | IS30 |  | Lactobacillus plantarum | 858 | 0 |
| FAM21835 | ISPp1 | IS30 |  | Pediococcus pentosaceus | 811 | 0 |
| FAM21838 | ISLdl3 | IS30 |  | Lactobacillus delbrueckii | 2284 | 0 |
| FAM21838 | IS1310 | IS256 |  | Enterococcus hirae | 2236 | 0 |
| FAM21838 | ISLpl1 | IS30 |  | Lactobacillus plantarum | 1176 | 0 |
| FAM21838 | ISPp1 | IS30 |  | Pediococcus pentosaceus | 1120 | 0 |
| FAM21838 | IS153 | IS3 | IS3 | Lactobacillus sanfranciscensis | 1047 | 0 |
| FAM23163 | ISLsa1 | IS30 |  | Lactobacillus sakei | 2042 | 0 |
| FAM23163 | ISLpl1 | IS30 |  | Lactobacillus plantarum | 1846 | 0 |
| FAM23163 | ISPp1 | IS30 |  | Pediococcus pentosaceus | 1742 | 0 |
| FAM23163 | IS153 | IS3 | IS3 | Lactobacillus sanfranciscensis | 1055 | 0 |
| FAM23164 | IS1165 | ISL3 |  | Leuconostoc mesenteroides | 2904 | 0 |
| FAM23164 | IS1310 | IS256 |  | Enterococcus hirae | 2292 | 0 |
| FAM23164 | IS153 | IS3 | IS3 | Lactobacillus sanfranciscensis | 1088 | 0 |
| FAM23164 | ISLpl1 | IS30 |  | Lactobacillus plantarum | 1059 | 0 |
| FAM23164 | ISPp1 | IS30 |  | Pediococcus pentosaceus | 1051 | 0 |
| FAM23165 | IS1165 | ISL3 |  | Leuconostoc mesenteroides | 2904 | 0 |
| FAM23165 | IS1310 | IS256 |  | Enterococcus hirae | 2089 | 0 |
| FAM23165 | ISLpl1 | IS30 |  | Lactobacillus plantarum | 1576 | 0 |
| FAM23165 | ISPp1 | IS30 |  | Pediococcus pentosaceus | 1520 | 0 |
| FAM23165 | IS153 | IS3 | IS3 | Lactobacillus sanfranciscensis | 1088 | 0 |
| FAM23166 | IS1165 | ISL3 |  | Leuconostoc mesenteroides | 2904 | 0 |
| FAM23166 | IS1310 | IS256 |  | Enterococcus hirae | 2300 | 0 |
| FAM23166 | ISLpl1 | IS30 |  | Lactobacillus plantarum | 1580 | 0 |
| FAM23166 | ISPp1 | IS30 |  | Pediococcus pentosaceus | 1524 | 0 |
| FAM23166 | IS153 | IS3 | IS3 | Lactobacillus sanfranciscensis | 1088 | 0 |
| FAM23168 | ISLsa1 | IS30 |  | Lactobacillus sakei | 2042 | 0 |
| FAM23168 | ISLpl1 | IS30 |  | Lactobacillus plantarum | 1846 | 0 |
| FAM23168 | ISPp1 | IS30 |  | Pediococcus pentosaceus | 1742 | 0 |
| FAM23168 | IS153 | IS3 | IS3 | Lactobacillus sanfranciscensis | 1031 | 0 |
| FAM23169 | ISLpl3 | IS5 | IS427 | Lactobacillus plantarum | 1635 | 0 |
| FAM23169 | IS1165 | ISL3 |  | Leuconostoc mesenteroides | 1536 | 0 |
| FAM23169 | IS1310 | IS256 |  | Enterococcus hirae | 1326 | 0 |
| FAM23169 | ISLpl1 | IS30 |  | Lactobacillus plantarum | 1314 | 0 |
| FAM23169 | ISPp1 | IS30 |  | Pediococcus pentosaceus | 1251 | 0 |
| FAM23169 | IS153 | IS3 | IS3 | Lactobacillus sanfranciscensis | 831 | 0 |
| FAM23279 | IS1165 | ISL3 |  | Leuconostoc mesenteroides | 2954 | 0 |
| FAM23279 | ISLdl3 | IS30 |  | Lactobacillus delbrueckii | 2284 | 0 |
| FAM23279 | ISLsa1 | IS30 |  | Lactobacillus sakei | 2024 | 0 |
| FAM23279 | IS1310 | IS256 |  | Enterococcus hirae | 1727 | 0 |
| FAM23279 | ISLpl1 | IS30 |  | Lactobacillus plantarum | 1639 | 0 |
| FAM23279 | ISLhe30 | IS30 |  | Lactobacillus helveticus | 1592 | 0 |
| FAM23279 | ISPp1 | IS30 |  | Pediococcus pentosaceus | 1544 | 0 |
| FAM23279 | ISLpl3 | IS5 | IS427 | Lactobacillus plantarum | 1509 | 0 |
| FAM23279 | IS153 | IS3 | IS3 | Lactobacillus sanfranciscensis | 1070 | 0 |
| FAM23279 | IS1297 | IS6 |  | Leuconostoc mesenteroides | 821 | 0 |
| FAM23279 | ISS1N | IS6 |  | Lactococcus lactis | 805 | 0 |
| FAM23279 | ISS1M | IS6 |  | Lactococcus lactis | 797 | 0 |
| FAM23279 | ISS1E | IS6 |  | Lactococcus lactis | 781 | 0 |
| FAM23279 | ISS1D | IS6 |  | Lactococcus lactis | 765 | 0 |
| FAM23279 | ISS1CH | IS6 |  | Lactococcus lactis | 729 | 0 |
| FAM23280 | IS1165 | ISL3 |  | Leuconostoc mesenteroides | 2954 | 0 |
| FAM23280 | ISLdl3 | IS30 |  | Lactobacillus delbrueckii | 2089 | 0 |
| FAM23280 | IS1310 | IS256 |  | Enterococcus hirae | 1887 | 0 |
| FAM23280 | ISLpl1 | IS30 |  | Lactobacillus plantarum | 1639 | 0 |
| FAM23280 | ISLhe30 | IS30 |  | Lactobacillus helveticus | 1592 | 0 |
| FAM23280 | ISPp1 | IS30 |  | Pediococcus pentosaceus | 1544 | 0 |
| FAM23280 | ISLpl3 | IS5 | IS427 | Lactobacillus plantarum | 1511 | 0 |
| FAM23280 | IS153 | IS3 | IS3 | Lactobacillus sanfranciscensis | 1070 | 0 |
| FAM23281 | IS1165 | ISL3 |  | Leuconostoc mesenteroides | 2954 | 0 |
| FAM23281 | ISLdl3 | IS30 |  | Lactobacillus delbrueckii | 2284 | 0 |
| FAM23281 | ISLsa1 | IS30 |  | Lactobacillus sakei | 2026 | 0 |
| FAM23281 | IS1310 | IS256 |  | Enterococcus hirae | 1945 | 0 |
| FAM23281 | ISLpl1 | IS30 |  | Lactobacillus plantarum | 1639 | 0 |
| FAM23281 | ISLhe30 | IS30 |  | Lactobacillus helveticus | 1592 | 0 |
| FAM23281 | ISPp1 | IS30 |  | Pediococcus pentosaceus | 1544 | 0 |
| FAM23281 | ISLpl3 | IS5 | IS427 | Lactobacillus plantarum | 1507 | 0 |
| FAM23281 | IS153 | IS3 | IS3 | Lactobacillus sanfranciscensis | 1070 | 0 |
| FAM23281 | IS1297 | IS6 |  | Leuconostoc mesenteroides | 821 | 0 |
| FAM23281 | ISS1N | IS6 |  | Lactococcus lactis | 805 | 0 |
| FAM23281 | ISS1M | IS6 |  | Lactococcus lactis | 797 | 0 |
| FAM23281 | ISS1E | IS6 |  | Lactococcus lactis | 781 | 0 |
| FAM23281 | ISS1D | IS6 |  | Lactococcus lactis | 765 | 0 |
| FAM23281 | ISS1CH | IS6 |  | Lactococcus lactis | 729 | 0 |
| FA23282 | IS1165 | ISL3 |  | Leuconostoc mesenteroides | 2926 | 0 |
| FA23282 | IS1163 | IS3 | IS3 | Lactobacillus sake | 2292 | 0 |
| FA23282 | ISLdl3 | IS30 |  | Lactobacillus delbrueckii | 2284 | 0 |
| FA23282 | IS1310 | IS256 |  | Enterococcus hirae | 2204 | 0 |
| FA23282 | ISLpl1 | IS30 |  | Lactobacillus plantarum | 2004 | 0 |
| FA23282 | ISPp1 | IS30 |  | Pediococcus pentosaceus | 1901 | 0 |
| FA23282 | IS153 | IS3 | IS3 | Lactobacillus sanfranciscensis | 1027 | 0 |
| IPLA 11117 | IS1165 | ISL3 |  | Leuconostoc mesenteroides | 2968 | 0 |
| IPLA 11117 | ISLfr1 | ISL3 |  | Lactobacillus fructivorans | 2829 | 0 |
| IPLA 11117 | ISLsa1 | IS30 |  | Lactobacillus sakei | 2026 | 0 |
| IPLA 11117 | ISLpl4 | IS982 |  | Lactobacillus plantarum | 1921 | 0 |
| IPLA 11117 | IS1310 | IS256 |  | Enterococcus hirae | 1852 | 0 |
| IPLA 11117 | ISLhe30 | IS30 |  | Lactobacillus helveticus | 1637 | 0 |
| IPLA 11117 | ISLpl1 | IS30 |  | Lactobacillus plantarum | 1600 | 0 |
| IPLA 11117 | ISPp1 | IS30 |  | Pediococcus pentosaceus | 1544 | 0 |
| IPLA 11117 | IS153 | IS3 | IS3 | Lactobacillus sanfranciscensis | 1070 | 0 |
| IPLA 11122 | ISLpl3 | IS5 | IS427 | Lactobacillus plantarum | 1628 | 0 |
| IPLA 11122 | IS153 | IS3 | IS3 | Lactobacillus sanfranciscensis | 831 | 0 |
| IPLA 11125 | IS1310 | IS256 |  | Enterococcus hirae | 2331 | 0 |
| IPLA 11125 | ISLpl1 | IS30 |  | Lactobacillus plantarum | 1957 | 0 |
| IPLA 11125 | ISPp1 | IS30 |  | Pediococcus pentosaceus | 1853 | 0 |
| IPLA 11129 | ISLdl3 | IS30 |  | Lactobacillus delbrueckii | 2268 | 0 |
| IPLA 11129 | ISLpl1 | IS30 |  | Lactobacillus plantarum | 1723 | 0 |
| IPLA 11129 | ISPp1 | IS30 |  | Pediococcus pentosaceus | 1637 | 0 |
| IPLA 11150 | IS1310 | IS256 |  | Enterococcus hirae | 2212 | 0 |
| IPLA 11150 | ISLsa1 | IS30 |  | Lactobacillus sakei | 2034 | 0 |
| IPLA 11150 | ISLpl4 | IS982 |  | Lactobacillus plantarum | 1921 | 0 |
| IPLA 11150 | ISLpl1 | IS30 |  | Lactobacillus plantarum | 1774 | 0 |
| IPLA 11150 | ISPp1 | IS30 |  | Pediococcus pentosaceus | 1703 | 0 |
| IPLA 11150 | IS1165 | ISL3 |  | Leuconostoc mesenteroides | 1622 | 0 |
| IPLA 11150 | IS153 | IS3 | IS3 | Lactobacillus sanfranciscensis | 1070 | 0 |
| IPLA 11151 | IS1310 | IS256 |  | Enterococcus hirae | 2212 | 0 |
| IPLA 11151 | ISLpl1 | IS30 |  | Lactobacillus plantarum | 1996 | 0 |
| IPLA 11151 | ISLpl4 | IS982 |  | Lactobacillus plantarum | 1921 | 0 |
| IPLA 11151 | ISPp1 | IS30 |  | Pediococcus pentosaceus | 1893 | 0 |
| IPLA 11151 | IS1165 | ISL3 |  | Leuconostoc mesenteroides | 1622 | 0 |
| IPLA 11151 | IS153 | IS3 | IS3 | Lactobacillus sanfranciscensis | 1070 | 0 |
| KEM | ISLpl3 | IS5 | IS427 | Lactobacillus plantarum | 1628 | 0 |
| KEM | IS153 | IS3 | IS3 | Lactobacillus sanfranciscensis | 952 | 0 |
| NBRC107865 | IS1165 | ISL3 |  | Leuconostoc mesenteroides | 2960 | 0 |
| NBRC107865 | IS1310 | IS256 |  | Enterococcus hirae | 2173 | 0 |
| NBRC107865 | IS153 | IS3 | IS3 | Lactobacillus sanfranciscensis | 815 | 0 |
| NSMJ16 | ISLsa1 | IS30 |  | Lactobacillus sakei | 1570 | 0 |
| NSMJ16 | ISLpl1 | IS30 |  | Lactobacillus plantarum | 1546 | 0 |
| NSMJ16 | ISPp1 | IS30 |  | Pediococcus pentosaceus | 1475 | 0 |
| NSMJ16 | IS153 | IS3 | IS3 | Lactobacillus sanfranciscensis | 1070 | 0 |
| NSMJ16 | IS1201 | IS256 |  | Lactobacillus helveticus | 878 | 0 |
| DSM15352 | ISLsa1 | IS30 |  | Lactobacillus sakei | 1903 | 0 |
| DSM15352 | IS1310 | IS256 |  | Enterococcus hirae | 1308 | 0 |
| DSM15352 | IS1165 | ISL3 |  | Leuconostoc mesenteroides | 1275 | 0 |
| DSM15352 | ISSsu5 | IS1380 |  | Streptococcus suis | 872 | 0 |
| DSM15352 | IS153 | IS3 | IS3 | Lactobacillus sanfranciscensis | 831 | 0 |

**Table S4.** Putative plasmids identified in *L. parabuchneri* genomes

| Strain | Identity | p-value | Accession Number | Topology | Length (bp) |
| --- | --- | --- | --- | --- | --- |
| NBRC107865 | 1.00 | 0 | NC_002123.1 | circular | 2295 |
| DSM5707 | 1.00 | 0 | NC_002123.1 | circular | 2295 |
| FAM23169 | 1.00 | 0 | NC_016635.1 | circular | 1815 |
| IPLA11150 | 1.00 | 0 | NC_016635.1 | circular | 1815 |
| IPLA11151 | 1.00 | 0 | NC_016635.1 | circular | 1815 |
| FAM21823 | 1.00 | 0 | NZ_CP017265.1 | circular | 28557 |
| FAM21731 | 1.00 | 0 | NZ_CP018797.1 | circular | 58093 |
| FAM21731 | 1.00 | 0 | NZ_CP018798.1 | circular | 67905 |
| FAM21829 | 1.00 | 0 | NZ_CP018798.1 | circular | 67905 |
| DSM15352 | 0.99 | 0 | NZ_CP047122.1 | linear | 42732 |
| NSMJ16 | 1.00 | 0 | NZ_CP050494.1 | circular | 55903 |
| NSMJ16 | 1.00 | 0 | NZ_CP050495.1 | circular | 43430 |
| NSMJ16 | 1.00 | 0 | NZ_CP050496.1 | circular | 37160 |
| IPLA11129 | 0.99 | 0 | NZ_CP065817.1 | circular | 2745 |
| FAM21834 | 1.00 | 0 | NZ_LM651913.1 | circular | 3369 |

**Table S5.** CRISPR elements and Cas clusters of twenty-eight *L. parabuchneri* strains.

| Strain | Element | CRISPR Id / Cas Type | Start | End | Spacer / Gene | Direction | Evidence Level |
| --- | --- | --- | --- | --- | --- | --- | --- |
| DSM15352 | Cas cluster | CAS-TypeIIA | 2582712 | 2588814 | 4 |  |  |
| DSM15352 | CRISPR | Lparabuchneri_DSM15352_1 | 71583 | 71739 | 1 | ND | 1 |
| DSM15352 | CRISPR | Lparabuchneri_DSM15352_2 | 2501891 | 2502916 | 15 | ND | 4 |
| DSM15352 | CRISPR | Lparabuchneri_DSM15352_3 | 2572251 | 2572354 | 1 | ND | 1 |
| DSM5707 | Cas cluster | CAS-TypeIE | 1286386 | 1295905 | 7 |  |  |
| DSM5707 | CRISPR | Lparabuchneri_DSM5707_1 | 1059711 | 1059811 | 1 | ND | 1 |
| DSM5707 | CRISPR | Lparabuchneri_DSM5707_2 | 1256380 | 1256652 | 4 | ND | 4 |
| DSM5707 | CRISPR | Lparabuchneri_DSM5707_3 | 1282644 | 1282853 | 3 | ND | 1 |
| DSM5707 | CRISPR | Lparabuchneri_DSM5707_4 | 1284236 | 1284446 | 3 | - | 1 |
| DSM5707 | CRISPR | Lparabuchneri_DSM5707_5 | 1295935 | 1296201 | 4 | ND | 3 |
| DSM5707 | CRISPR | Lparabuchneri_DSM5707_6 | 1450226 | 1450626 | 6 | ND | 4 |
| DSM5707 | CRISPR | Lparabuchneri_DSM5707_7 | 1468916 | 1469019 | 1 | ND | 1 |
| FAM21731 | Cas cluster | CAS-TypeIE | 953895 | 963414 | 7 |  |  |
| FAM21731 | CRISPR | Lparabuchneri_FAM21731_1 | 925473 | 925805 | 5 | ND | 3 |
| FAM21731 | CRISPR | Lparabuchneri_FAM21731_2 | 953660 | 953865 | 3 | ND | 1 |
| FAM21731 | CRISPR | Lparabuchneri_FAM21731_3 | 965354 | 965564 | 3 | ND | 1 |
| FAM21731 | CRISPR | Lparabuchneri_FAM21731_4 | 966947 | 967156 | 3 | ND | 1 |
| FAM21731 | CRISPR | Lparabuchneri_FAM21731_5 | 993149 | 993604 | 7 | ND | 4 |
| FAM21809 | Cas cluster | CAS-TypeIE | 957887 | 967406 | 7 |  |  |
| FAM21809 | CRISPR | Lparabuchneri_FAM21809_1 | 929137 | 929470 | 5 | ND | 4 |
| FAM21809 | CRISPR | Lparabuchneri_FAM21809_2 | 957591 | 957857 | 4 | ND | 3 |
| FAM21809 | CRISPR | Lparabuchneri_FAM21809_3 | 969346 | 969556 | 3 | ND | 1 |
| FAM21809 | CRISPR | Lparabuchneri_FAM21809_4 | 970939 | 971148 | 3 | ND | 1 |
| FAM21809 | CRISPR | Lparabuchneri_FAM21809_5 | 997141 | 997719 | 9 | ND | 4 |
| FAM21809 | CRISPR | Lparabuchneri_FAM21809_6 | 1284810 | 1284891 | 1 | ND | 1 |
| FAM21809 | CRISPR | Lparabuchneri_FAM21809_7 | 1609445 | 1609535 | 1 | ND | 1 |
| FAM21823 | Cas cluster | CAS-TypeIE | 992969 | 1002488 | 7 |  |  |
| FAM21823 | CRISPR | Lparabuchneri_FAM21823_1 | 965087 | 965420 | 5 | ND | 4 |
| FAM21823 | CRISPR | Lparabuchneri_FAM21823_2 | 992673 | 992939 | 4 | ND | 3 |
| FAM21823 | CRISPR | Lparabuchneri_FAM21823_3 | 1004428 | 1004638 | 3 | ND | 1 |
| FAM21823 | CRISPR | Lparabuchneri_FAM21823_4 | 1006021 | 1006230 | 3 | ND | 1 |
| FAM21823 | CRISPR | Lparabuchneri_FAM21823_5 | 1032222 | 1032739 | 8 | ND | 4 |
| FAM21823 | CRISPR | Lparabuchneri_FAM21823_6 | 2655962 | 2656063 | 1 | ND | 1 |
| FAM21829 | Cas cluster | CAS-TypeIE | 825223 | 834742 | 7 |  |  |
| FAM21829 | CRISPR | Lparabuchneri_FAM21829_1 | 797541 | 797935 | 6 | ND | 4 |
| FAM21829 | CRISPR | Lparabuchneri_FAM21829_2 | 824927 | 825193 | 4 | ND | 3 |
| FAM21829 | CRISPR | Lparabuchneri_FAM21829_3 | 836682 | 836892 | 3 | ND | 1 |
| FAM21829 | CRISPR | Lparabuchneri_FAM21829_4 | 838275 | 838484 | 3 | ND | 1 |
| FAM21829 | CRISPR | Lparabuchneri_FAM21829_5 | 865676 | 865948 | 4 | ND | 4 |
| FAM21834 | Cas cluster | CAS-TypeIE | 895490 | 905009 | 7 |  |  |
| FAM21834 | CRISPR | Lparabuchneri_FAM21834_1 | 860238 | 860632 | 6 | ND | 4 |
| FAM21834 | CRISPR | Lparabuchneri_FAM21834_2 | 906949 | 907159 | 3 | ND | 1 |
| FAM21834 | CRISPR | Lparabuchneri_FAM21834_3 | 908542 | 908751 | 3 | ND | 1 |
| FAM21834 | CRISPR | Lparabuchneri_FAM21834_4 | 934743 | 935199 | 7 | ND | 4 |
| FAM21834 | CRISPR | Lparabuchneri_FAM21834_5 | 2667760 | 2667863 | 1 | ND | 1 |
| FAM21834 | CRISPR | Lparabuchneri_FAM21834_6 | 2698940 | 2699043 | 1 | ND | 1 |
| FAM21835 | Cas cluster | CAS-TypeIE | 866471 | 875914 | 7 |  |  |
| FAM21835 | CRISPR | Lparabuchneri_FAM21835_1 | 865804 | 866441 | 10 | ND | 4 |
| FAM21835 | CRISPR | Lparabuchneri_FAM21835_2 | 879739 | 879950 | 3 | ND | 1 |
| FAM21835 | CRISPR | Lparabuchneri_FAM21835_3 | 891618 | 892072 | 7 | ND | 4 |
| FAM21835 | CRISPR | Lparabuchneri_FAM21835_4 | 1494202 | 1494292 | 1 | ND | 1 |
| FAM21835 | CRISPR | Lparabuchneri_FAM21835_5 | 2539467 | 2539568 | 1 | ND | 1 |
| FAM21835 | CRISPR | Lparabuchneri_FAM21835_6 | 2562052 | 2562208 | 1 | ND | 1 |
| FAM21838 | Cas cluster | CAS-TypeIE | 819502 | 829230 | 7 |  |  |
| FAM21838 | CRISPR | Lparabuchneri_FAM21838_1 | 818835 | 819472 | 10 | ND | 4 |
| FAM21838 | CRISPR | Lparabuchneri_FAM21838_2 | 833054 | 833328 | 4 | ND | 3 |
| FAM21838 | CRISPR | Lparabuchneri_FAM21838_3 | 844996 | 845633 | 10 | ND | 4 |
| FAM21838 | CRISPR | Lparabuchneri_FAM21838_4 | 1126345 | 1126426 | 1 | ND | 1 |
| FAM21838 | CRISPR | Lparabuchneri_FAM21838_5 | 1450322 | 1450412 | 1 | ND | 1 |
| FAM23163 | Cas cluster | CAS-TypeIE | 831386 | 840905 | 7 |  |  |
| FAM23163 | CRISPR | Lparabuchneri_FAM23163_1 | 803704 | 804098 | 6 | ND | 4 |
| FAM23163 | CRISPR | Lparabuchneri_FAM23163_2 | 831090 | 831356 | 4 | ND | 3 |
| FAM23163 | CRISPR | Lparabuchneri_FAM23163_3 | 842845 | 843055 | 3 | ND | 1 |
| FAM23163 | CRISPR | Lparabuchneri_FAM23163_4 | 844438 | 844647 | 3 | ND | 1 |
| FAM23163 | CRISPR | Lparabuchneri_FAM23163_5 | 871676 | 871948 | 4 | ND | 4 |
| FAM23163 | CRISPR | Lparabuchneri_FAM23163_6 | 2513339 | 2513495 | 1 | ND | 1 |
| FAM23163 | CRISPR | Lparabuchneri_FAM23163_7 | 2542460 | 2542561 | 1 | ND | 1 |
| FAM23164 | Cas cluster | CAS-TypeIE | 938679 | 948198 | 7 |  |  |
| FAM23164 | CRISPR | Lparabuchneri_FAM23164_1 | 909929 | 910262 | 5 | ND | 4 |
| FAM23164 | CRISPR | Lparabuchneri_FAM23164_2 | 938383 | 938649 | 4 | ND | 3 |
| FAM23164 | CRISPR | Lparabuchneri_FAM23164_3 | 950138 | 950348 | 3 | ND | 1 |
| FAM23164 | CRISPR | Lparabuchneri_FAM23164_4 | 951731 | 951940 | 3 | ND | 1 |
| FAM23164 | CRISPR | Lparabuchneri_FAM23164_5 | 977932 | 978510 | 9 | ND | 4 |
| FAM23164 | CRISPR | Lparabuchneri_FAM23164_6 | 1268295 | 1268376 | 1 | ND | 1 |
| FAM23164 | CRISPR | Lparabuchneri_FAM23164_7 | 1593113 | 1593203 | 1 | ND | 1 |
| FAM23164 | CRISPR | Lparabuchneri_FAM23164_8 | 2627217 | 2627320 | 1 | ND | 1 |
| FAM23165 | Cas cluster | CAS-TypeIE | 968074 | 977593 | 7 |  |  |
| FAM23165 | CRISPR | Lparabuchneri_FAM23165_1 | 548577 | 548734 | 1 | ND | 1 |
| FAM23165 | CRISPR | Lparabuchneri_FAM23165_2 | 939324 | 939657 | 5 | ND | 4 |
| FAM23165 | CRISPR | Lparabuchneri_FAM23165_3 | 967778 | 968044 | 4 | ND | 3 |
| FAM23165 | CRISPR | Lparabuchneri_FAM23165_4 | 979533 | 979743 | 3 | ND | 1 |
| FAM23165 | CRISPR | Lparabuchneri_FAM23165_5 | 981126 | 981335 | 3 | ND | 1 |
| FAM23165 | CRISPR | Lparabuchneri_FAM23165_6 | 1007327 | 1007905 | 9 | ND | 4 |
| FAM23165 | CRISPR | Lparabuchneri_FAM23165_7 | 1297695 | 1297776 | 1 | ND | 1 |
| FAM23165 | CRISPR | Lparabuchneri_FAM23165_8 | 1622513 | 1622603 | 1 | ND | 1 |
| FAM23166 | Cas cluster | CAS-TypeIE | 915006 | 924525 | 7 |  |  |
| FAM23166 | CRISPR | Lparabuchneri_FAM23166_1 | 886867 | 887200 | 5 | ND | 4 |
| FAM23166 | CRISPR | Lparabuchneri_FAM23166_2 | 914710 | 914976 | 4 | ND | 3 |
| FAM23166 | CRISPR | Lparabuchneri_FAM23166_3 | 926465 | 926675 | 3 | ND | 1 |
| FAM23166 | CRISPR | Lparabuchneri_FAM23166_4 | 928058 | 928267 | 3 | ND | 1 |
| FAM23166 | CRISPR | Lparabuchneri_FAM23166_5 | 954259 | 954837 | 9 | ND | 4 |
| FAM23166 | CRISPR | Lparabuchneri_FAM23166_6 | 1245759 | 1245840 | 1 | ND | 1 |
| FAM23166 | CRISPR | Lparabuchneri_FAM23166_7 | 1570581 | 1570671 | 1 | ND | 1 |
| FAM23166 | CRISPR | Lparabuchneri_FAM23166_8 | 2660055 | 2660158 | 1 | ND | 1 |
| FAM23168 | Cas cluster | CAS-TypeIE | 895737 | 905256 | 7 |  |  |
| FAM23168 | CRISPR | Lparabuchneri_FAM23168_1 | 868055 | 868449 | 6 | ND | 4 |
| FAM23168 | CRISPR | Lparabuchneri_FAM23168_2 | 895441 | 895707 | 4 | ND | 3 |
| FAM23168 | CRISPR | Lparabuchneri_FAM23168_3 | 907196 | 907406 | 3 | ND | 1 |
| FAM23168 | CRISPR | Lparabuchneri_FAM23168_4 | 908789 | 908998 | 3 | ND | 1 |
| FAM23168 | CRISPR | Lparabuchneri_FAM23168_5 | 936027 | 936299 | 4 | ND | 4 |
| FAM23168 | CRISPR | Lparabuchneri_FAM23168_6 | 2513102 | 2513258 | 1 | ND | 1 |
| FAM23168 | CRISPR | Lparabuchneri_FAM23168_7 | 2542299 | 2542400 | 1 | ND | 1 |
| FAM23169 | Cas cluster | CAS-TypeIE | 900993 | 910512 | 7 |  |  |
| FAM23169 | CRISPR | Lparabuchneri_FAM23169_1 | 873182 | 873576 | 6 | ND | 4 |
| FAM23169 | CRISPR | Lparabuchneri_FAM23169_2 | 900814 | 900963 | 2 | ND | 1 |
| FAM23169 | CRISPR | Lparabuchneri_FAM23169_3 | 912452 | 912662 | 3 | ND | 1 |
| FAM23169 | CRISPR | Lparabuchneri_FAM23169_4 | 914045 | 914254 | 3 | ND | 1 |
| FAM23169 | CRISPR | Lparabuchneri_FAM23169_5 | 940246 | 940702 | 7 | ND | 4 |
| FAM23169 | CRISPR | Lparabuchneri_FAM23169_6 | 2694688 | 2694789 | 1 | ND | 1 |
| FAM23279 | Cas cluster | CAS-TypeIE | 901956 | 911399 | 7 |  |  |
| FAM23279 | CRISPR | Lparabuchneri_FAM23279_1 | 901289 | 901926 | 10 | ND | 4 |
| FAM23279 | CRISPR | Lparabuchneri_FAM23279_2 | 915224 | 915435 | 3 | ND | 1 |
| FAM23279 | CRISPR | Lparabuchneri_FAM23279_3 | 927103 | 927618 | 8 | ND | 4 |
| FAM23279 | CRISPR | Lparabuchneri_FAM23279_4 | 1209541 | 1209622 | 1 | ND | 1 |
| FAM23279 | CRISPR | Lparabuchneri_FAM23279_5 | 1535554 | 1535644 | 1 | ND | 1 |
| FAM23279 | CRISPR | Lparabuchneri_FAM23279_6 | 2542541 | 2542644 | 1 | ND | 1 |
| FAM23280 | Cas cluster | CAS-TypeIE | 879321 | 888764 | 7 |  |  |
| FAM23280 | CRISPR | Lparabuchneri_FAM23280_1 | 878654 | 879291 | 10 | ND | 4 |
| FAM23280 | CRISPR | Lparabuchneri_FAM23280_2 | 892589 | 892800 | 3 | ND | 1 |
| FAM23280 | CRISPR | Lparabuchneri_FAM23280_3 | 904468 | 904983 | 8 | ND | 4 |
| FAM23280 | CRISPR | Lparabuchneri_FAM23280_4 | 1211423 | 1211504 | 1 | ND | 1 |
| FAM23280 | CRISPR | Lparabuchneri_FAM23280_5 | 1536070 | 1536160 | 1 | ND | 1 |
| FAM23280 | CRISPR | Lparabuchneri_FAM23280_6 | 2513574 | 2513731 | 1 | ND | 1 |
| FAM23281 | Cas cluster | CAS-TypeIE | 900477 | 909920 | 7 |  |  |
| FAM23281 | CRISPR | Lparabuchneri_FAM23281_1 | 899810 | 900447 | 10 | ND | 4 |
| FAM23281 | CRISPR | Lparabuchneri_FAM23281_2 | 913745 | 913956 | 3 | ND | 1 |
| FAM23281 | CRISPR | Lparabuchneri_FAM23281_3 | 925624 | 926139 | 8 | ND | 4 |
| FAM23281 | CRISPR | Lparabuchneri_FAM23281_4 | 1210504 | 1210585 | 1 | ND | 1 |
| FAM23281 | CRISPR | Lparabuchneri_FAM23281_5 | 1535884 | 1535974 | 1 | ND | 1 |
| FAM23281 | CRISPR | Lparabuchneri_FAM23281_6 | 2543567 | 2543670 | 1 | ND | 1 |
| FAM23282 | Cas cluster | CAS-TypeIE | 765994 | 775437 | 7 |  |  |
| FAM23282 | CRISPR | Lparabuchneri_FAM23282_1 | 765327 | 765964 | 10 | ND | 4 |
| FAM23282 | CRISPR | Lparabuchneri_FAM23282_2 | 779262 | 779473 | 3 | ND | 1 |
| FAM23282 | CRISPR | Lparabuchneri_FAM23282_3 | 791141 | 791656 | 8 | ND | 4 |
| FAM23282 | CRISPR | Lparabuchneri_FAM23282_4 | 1390730 | 1390820 | 1 | ND | 1 |
| FAM23282 | CRISPR | Lparabuchneri_FAM23282_5 | 2477601 | 2477750 | 1 | ND | 1 |
| FAM23282 | CRISPR | Lparabuchneri_FAM23282_6 | 2529537 | 2529640 | 1 | ND | 1 |
| FAM23282 | CRISPR | Lparabuchneri_FAM23282_7 | 2566460 | 2566563 | 1 | ND | 1 |
| IPLA11117 | Cas cluster | CAS-TypeIE | 847111 | 856630 | 7 |  |  |
| IPLA11117 | CRISPR | Lparabuchneri_IPLA11117_1 | 856660 | 856926 | 4 | ND | 3 |
| IPLA11117 | CRISPR | Lparabuchneri_IPLA11117_2 | 883918 | 884251 | 5 | ND | 4 |
| IPLA11117 | CRISPR | Lparabuchneri_IPLA11117_3 | 1909178 | 1909332 | 1 | ND | 1 |
| IPLA11117 | CRISPR | Lparabuchneri_IPLA11117_4 | 2167753 | 2167963 | 3 | ND | 1 |
| IPLA11117 | CRISPR | Lparabuchneri_IPLA11117_5 | 2169346 | 2169555 | 3 | ND | 1 |
| IPLA11117 | CRISPR | Lparabuchneri_IPLA11117_6 | 2195547 | 2195819 | 4 | ND | 4 |
| IPLA11117 | CRISPR | Lparabuchneri_IPLA11117_7 | 2327509 | 2327612 | 1 | ND | 1 |
| IPLA11122 | Cas cluster | CAS-TypeIE | 1612347 | 1621866 | 7 |  |  |
| IPLA11122 | CRISPR | Lparabuchneri_IPLA11122_1 | 1584665 | 1585059 | 6 | ND | 4 |
| IPLA11122 | CRISPR | Lparabuchneri_IPLA11122_2 | 1612051 | 1612317 | 4 | ND | 3 |
| IPLA11122 | CRISPR | Lparabuchneri_IPLA11122_3 | 1623806 | 1624016 | 3 | ND | 1 |
| IPLA11122 | CRISPR | Lparabuchneri_IPLA11122_4 | 1625399 | 1625608 | 3 | ND | 1 |
| IPLA11122 | CRISPR | Lparabuchneri_IPLA11122_5 | 1651600 | 1651872 | 4 | ND | 4 |
| IPLA11125 | Cas cluster | CAS-TypeIE | 1110596 | 1120115 | 7 |  |  |
| IPLA11125 | CRISPR | Lparabuchneri_IPLA11125_1 | 218389 | 218485 | 1 | ND | 1 |
| IPLA11125 | CRISPR | Lparabuchneri_IPLA11125_2 | 1052347 | 1052450 | 1 | ND | 1 |
| IPLA11125 | CRISPR | Lparabuchneri_IPLA11125_3 | 1082914 | 1083308 | 6 | ND | 4 |
| IPLA11125 | CRISPR | Lparabuchneri_IPLA11125_4 | 1110300 | 1110566 | 4 | ND | 3 |
| IPLA11125 | CRISPR | Lparabuchneri_IPLA11125_5 | 1122055 | 1122265 | 3 | ND | 1 |
| IPLA11125 | CRISPR | Lparabuchneri_IPLA11125_6 | 1123648 | 1123857 | 3 | ND | 1 |
| IPLA11125 | CRISPR | Lparabuchneri_IPLA11125_7 | 1149849 | 1150121 | 4 | ND | 4 |
| IPLA11129 | Cas cluster | CAS-TypeIE | 1447748 | 1452704 | 6 |  |  |
| IPLA11129 | CRISPR | Lparabuchneri_IPLA11129_1 | 365519 | 365728 | 3 | ND | 1 |
| IPLA11129 | CRISPR | Lparabuchneri_IPLA11129_2 | 367111 | 367321 | 3 | - | 1 |
| IPLA11129 | CRISPR | Lparabuchneri_IPLA11129_3 | 400517 | 401095 | 9 | ND | 4 |
| IPLA11129 | CRISPR | Lparabuchneri_IPLA11129_4 | 1111119 | 1111220 | 1 | ND | 1 |
| IPLA11129 | CRISPR | Lparabuchneri_IPLA11129_5 | 1419199 | 1419593 | 6 | ND | 4 |
| IPLA11129 | CRISPR | Lparabuchneri_IPLA11129_6 | 1447452 | 1447718 | 4 | ND | 3 |
| IPLA11150 | Cas cluster | CAS-TypeIE | 2103127 | 2112646 | 7 |  |  |
| IPLA11150 | CRISPR | Lparabuchneri_IPLA11150_1 | 1551129 | 1551286 | 1 | ND | 1 |
| IPLA11150 | CRISPR | Lparabuchneri_IPLA11150_2 | 1771270 | 1771664 | 6 | ND | 4 |
| IPLA11150 | CRISPR | Lparabuchneri_IPLA11150_3 | 1822544 | 1822647 | 1 | ND | 1 |
| IPLA11150 | CRISPR | Lparabuchneri_IPLA11150_4 | 2056100 | 2056255 | 1 | ND | 1 |
| IPLA11150 | CRISPR | Lparabuchneri_IPLA11150_5 | 2102831 | 2103097 | 4 | ND | 3 |
| IPLA11150 | CRISPR | Lparabuchneri_IPLA11150_6 | 2114586 | 2114796 | 3 | ND | 1 |
| IPLA11150 | CRISPR | Lparabuchneri_IPLA11150_7 | 2116179 | 2116388 | 3 | ND | 1 |
| IPLA11150 | CRISPR | Lparabuchneri_IPLA11150_8 | 2142380 | 2142652 | 4 | ND | 4 |
| IPLA11151 | Cas cluster | CAS-TypeIE | 2097293 | 2106812 | 7 |  |  |
| IPLA11151 | CRISPR | Lparabuchneri_IPLA11151_1 | 1435884 | 1436278 | 6 | ND | 4 |
| IPLA11151 | CRISPR | Lparabuchneri_IPLA11151_2 | 1455240 | 1455397 | 1 | ND | 1 |
| IPLA11151 | CRISPR | Lparabuchneri_IPLA11151_3 | 2067287 | 2067559 | 4 | ND | 4 |
| IPLA11151 | CRISPR | Lparabuchneri_IPLA11151_4 | 2093551 | 2093760 | 3 | ND | 1 |
| IPLA11151 | CRISPR | Lparabuchneri_IPLA11151_5 | 2095143 | 2095353 | 3 | - | 1 |
| IPLA11151 | CRISPR | Lparabuchneri_IPLA11151_6 | 2106842 | 2107108 | 4 | ND | 3 |
| KEM | Cas cluster | CAS-TypeIE | 957325 | 966844 | 7 |  |  |
| KEM | CRISPR | Lparabuchneri_KEM_1 | 929767 | 930099 | 5 | ND | 3 |
| KEM | CRISPR | Lparabuchneri_KEM_2 | 957090 | 957295 | 3 | ND | 1 |
| KEM | CRISPR | Lparabuchneri_KEM_3 | 968784 | 968994 | 3 | ND | 1 |
| KEM | CRISPR | Lparabuchneri_KEM_4 | 970377 | 970586 | 3 | ND | 1 |
| KEM | CRISPR | Lparabuchneri_KEM_5 | 996577 | 996851 | 4 | ND | 4 |
| NBRC107865 | Cas cluster | CAS-TypeIE | 1607797 | 1617316 | 7 |  |  |
| NBRC107865 | CRISPR | Lparabuchneri_NBRC107865_1 | 416591 | 416863 | 4 | ND | 4 |
| NBRC107865 | CRISPR | Lparabuchneri_NBRC107865_2 | 1607501 | 1607767 | 4 | ND | 3 |
| NBRC107865 | CRISPR | Lparabuchneri_NBRC107865_3 | 1619256 | 1619466 | 3 | ND | 1 |
| NBRC107865 | CRISPR | Lparabuchneri_NBRC107865_4 | 1620849 | 1621058 | 3 | ND | 1 |
| NBRC107865 | CRISPR | Lparabuchneri_NBRC107865_5 | 2302197 | 2302591 | 6 | ND | 4 |
| NBRC107865 | CRISPR | Lparabuchneri_NBRC107865_6 | 2428460 | 2428617 | 1 | ND | 1 |
| NBRC107865 | CRISPR | Lparabuchneri_NBRC107865_7 | 2537167 | 2537261 | 1 | ND | 1 |
| NSMJ16 | Cas cluster | CAS-TypeIE | 1012902 | 1022421 | 7 |  |  |
| NSMJ16 | CRISPR | Lparabuchneri_NSMJ16_1 | 981517 | 982095 | 9 | ND | 4 |
| NSMJ16 | CRISPR | Lparabuchneri_NSMJ16_2 | 1009160 | 1009369 | 3 | ND | 1 |
| NSMJ16 | CRISPR | Lparabuchneri_NSMJ16_3 | 1010752 | 1010962 | 3 | - | 1 |
| NSMJ16 | CRISPR | Lparabuchneri_NSMJ16_4 | 1022451 | 1022717 | 4 | ND | 3 |
| NSMJ16 | CRISPR | Lparabuchneri_NSMJ16_5 | 2534747 | 2534904 | 1 | ND | 1 |

**Table S6.** Grouping of *L. parabuchneri* based on CRISPR loci, spacer identity, and repeat identity.

| **Strain** | **CRISPR locus** | **Group by spacer identity** | **Group by repeat identity** |
| --- | --- | --- | --- |
| DSM15352 | 1 |  |  |
| DSM5707 | 1 | 1 | 1 |
| DSM5707 | 2 |  | 8 |
| FAM21731 | 1 | 2 | 4 |
| FAM21731 | 2 |  |  |
| FAM21809 | 1 | 4 | 3 |
| FAM21809 | 2 | 7 | 7 |
| FAM21823 | 1 | 4 | 3 |
| FAM21823 | 2 |  |  |
| FAM21829 | 1 | 3 | 2 |
| FAM21829 | 2 | 5 | 5 |
| FAM21834 | 1 | 3 | 2 |
| FAM21834 | 2 | 6 | 6 |
| FAM21835 | 1 | 10 | 10 |
| FAM21835 | 2 |  |  |
| FAM21838 | 1 | 10 | 10 |
| FAM21838 | 2 |  |  |
| FAM23163 | 1 | 3 | 2 |
| FAM23163 | 2 | 5 | 5 |
| FAM23164 | 1 | 4 | 3 |
| FAM23164 | 2 | 7 | 7 |
| FAM23165 | 1 | 4 | 3 |
| FAM23165 | 2 | 7 | 7 |
| FAM23166 | 1 | 4 | 3 |
| FAM23166 | 2 | 7 | 7 |
| FAM23168 | 1 | 3 | 2 |
| FAM23168 | 2 | 5 | 5 |
| FAM23169 | 1 | 3 | 2 |
| FAM23169 | 2 | 6 | 6 |
| FAM23279 | 1 | 10 | 10 |
| FAM23279 | 2 | 9 | 9 |
| FAM23280 | 1 | 10 | 10 |
| FAM23280 | 2 | 9 | 9 |
| FAM23281 | 1 | 10 | 10 |
| FAM23281 | 2 | 9 | 9 |
| FAM23282 | 1 | 10 | 10 |
| FAM23282 | 2 | 9 | 9 |
| IPLA11117 | 1 |  |  |
| IPLA11117 | 2 | 5 |  |
| IPLA11122 | 1 | 3 | 2 |
| IPLA11122 | 2 | 5 | 5 |
| IPLA11125 | 1 | 3 | 2 |
| IPLA11125 | 2 | 5 | 5 |
| IPLA11129 | 1 | 7 |  |
| IPLA11129 | 2 | 3 | 2 |
| IPLA11150 | 1 | 8 | 8 |
| IPLA11150 | 2 | 5 | 5 |
| IPLA11151 | 1 | 8 | 8 |
| IPLA11151 | 2 | 1 | 1 |
| KEM | 1 |  | 4 |
| KEM | 2 | 2 |  |
| NBRC107865 | 1 | 1 | 1 |
| NBRC107865 | 2 | 3 | 2 |
| NSMJ16 | 1 |  |  |

**Table S7**. Presence (1) and absence (0) of genes functional in lactate to 1,2-propanediol metabolism, arginine metabolism, and histidine to histamine conversion in twenty-eight *L. parabuchn*eri.

| **Strain** | **D-lactate dehydrogenase [EC:1.1.1.28]** | **L-lactate dehydrogenase [EC:1.1.1.27]** | **Lactaldehyde reductase** | **Lactaldehyde dehydrogenase [EC:1.2.1.22]** | **Arginine deiminase [EC:3.5.3.6]** | **Ornithine carbamoyltransferase [EC:2.1.3.3]** | **Carbamate kinase [EC:2.7.2.2]** | **Arginine/ornithine antiporter** | **Histidine decarboxylase (hdcA)** | **Histidine decarboxylase maturation protein (hdcB)** | **Histidine trna ligase (hisS)** | **Histidine/histamine antiporter (hdcC)** |
| --- | --- | --- | --- | --- | --- | --- | --- | --- | --- | --- | --- | --- |
| DSM15352 | 1 | 1 | 1 | 1 | 1 | 1 | 1 | 1 | 0 | 0 | 1 | 0 |
| DSM5707 | 1 | 1 | 1 | 1 | 1 | 1 | 1 | 1 | 1 | 1 | 1 | 1 |
| FAM21731 | 1 | 1 | 1 | 1 | 1 | 1 | 1 | 1 | 1 | 1 | 1 | 1 |
| FAM21809 | 1 | 1 | 1 | 1 | 1 | 1 | 1 | 1 | 1 | 1 | 1 | 1 |
| FAM21823 | 1 | 1 | 1 | 1 | 1 | 1 | 1 | 1 | 1 | 1 | 1 | 1 |
| FAM21829 | 1 | 1 | 1 | 1 | 1 | 1 | 1 | 1 | 1 | 1 | 1 | 1 |
| FAM21834 | 1 | 1 | 1 | 1 | 1 | 1 | 1 | 1 | 1 | 1 | 1 | 1 |
| FAM21835 | 1 | 1 | 1 | 1 | 1 | 1 | 1 | 1 | 0 | 0 | 1 | 0 |
| FAM21838 | 1 | 1 | 1 | 1 | 1 | 1 | 1 | 1 | 0 | 0 | 1 | 0 |
| FAM23163 | 1 | 1 | 1 | 1 | 1 | 1 | 1 | 1 | 1 | 1 | 1 | 1 |
| FAM23164 | 1 | 1 | 1 | 1 | 1 | 1 | 1 | 1 | 1 | 1 | 1 | 1 |
| FAM23165 | 1 | 1 | 1 | 1 | 1 | 1 | 1 | 1 | 1 | 1 | 1 | 1 |
| FAM23166 | 1 | 1 | 1 | 1 | 1 | 1 | 1 | 1 | 1 | 1 | 1 | 1 |
| FAM23168 | 1 | 1 | 1 | 1 | 1 | 1 | 1 | 1 | 1 | 1 | 1 | 1 |
| FAM23169 | 1 | 1 | 1 | 1 | 1 | 1 | 1 | 1 | 1 | 1 | 1 | 1 |
| FAM23279 | 1 | 1 | 1 | 1 | 1 | 1 | 1 | 1 | 0 | 0 | 1 | 0 |
| FAM23280 | 1 | 1 | 1 | 1 | 1 | 1 | 1 | 1 | 0 | 0 | 1 | 0 |
| FAM23281 | 1 | 1 | 1 | 1 | 1 | 1 | 1 | 1 | 0 | 0 | 1 | 0 |
| FAM23282 | 1 | 1 | 1 | 1 | 1 | 1 | 1 | 1 | 0 | 0 | 1 | 0 |
| IPLA11117 | 1 | 1 | 1 | 1 | 1 | 1 | 1 | 1 | 1 | 1 | 1 | 1 |
| IPLA11122 | 1 | 1 | 1 | 1 | 1 | 1 | 1 | 1 | 1 | 1 | 1 | 1 |
| IPLA11125 | 1 | 1 | 1 | 1 | 1 | 1 | 1 | 1 | 1 | 1 | 1 | 1 |
| IPLA11129 | 1 | 1 | 1 | 1 | 1 | 1 | 1 | 1 | 1 | 1 | 1 | 1 |
| IPLA11150 | 1 | 1 | 1 | 1 | 1 | 1 | 1 | 1 | 1 | 1 | 1 | 1 |
| IPLA11151 | 1 | 1 | 1 | 1 | 1 | 1 | 1 | 1 | 1 | 1 | 1 | 1 |
| KEM | 1 | 1 | 1 | 1 | 1 | 1 | 1 | 1 | 1 | 1 | 1 | 1 |
| NBRC107865 | 1 | 1 | 1 | 1 | 1 | 1 | 1 | 1 | 1 | 1 | 1 | 1 |
| NSMJ16 | 1 | 1 | 1 | 1 | 1 | 1 | 1 | 1 | 0 | 0 | 1 | 0 |
